# Sex differences in response to obesity and caloric restriction on cognition and hippocampal measures of autophagic-lysosomal transcripts and signaling pathways

**DOI:** 10.1186/s12868-023-00840-1

**Published:** 2024-01-02

**Authors:** Sadie B. Baer, Adrianah D. Dorn, Danielle M. Osborne

**Affiliations:** grid.415867.9 0000 0004 0456 1286 R.S. Dow Neurobiology, Legacy Research Institute, Portland, OR USA

**Keywords:** Obesity, Sex-differences, Autophagy, Caloric restriction, TFE3, Autophagy, Lysosomal degradation, Watermaze

## Abstract

**Background:**

Obesity rates in the U.S. continue to increase, with nearly 50% of the population being either obese or morbidly obese. Obesity, along with female sex, are leading risk factors for sporadic Alzheimer’s Disease (AD) necessitating the need to better understand how these variables impact cellular function independent of age or genetic mutations. Animal and clinical studies both indicate that autophagy-lysosomal pathway (ALP) dysfunction is among the earliest known cellular systems to become perturbed in AD, preceding cognitive decline, yet little is known about how obesity and sex affects these cellular functions in the hippocampus, a brain region uniquely susceptible to the negative effects of obesity. We hypothesized that obesity would negatively affect key markers of ALP in the hippocampus, effects would vary based on sex, and that caloric restriction would counteract obesity effects.

**Methods:**

Female and male mice were placed on an obesogenic diet for 10 months, at which point half were switched to caloric restriction for three months, followed by cognitive testing in the Morris watermaze. Hippocampus was analyzed by western blot and qPCR.

**Results:**

Cognitive function in female mice responded differently to caloric restriction based on whether they were on a normal or obesogenic diet; male cognition was only mildly affected by caloric restriction and not obesity. Significant male-specific changes occurred in cellular markers of autophagy, including obesity increasing pAkt, *Slc38a9*, and *Atg12*, while caloric restriction reduced pRPS6 and increased *Atg7*. In contrast females experienced changes due to diet/caloric restriction predominately in lysosomal markers including increased *TFE3*, *FLCN*, *FNIP2*, and pAMPK.

**Conclusions:**

Results support that hippocampal ALP is a target of obesity and that sex shapes molecular responses, while providing insight into how dietary manipulations affect learning and memory based on sex.

**Supplementary Information:**

The online version contains supplementary material available at 10.1186/s12868-023-00840-1.

## Introduction

Over 50% of Americans are obese or severely obese [1]. Given that obesity predisposes individuals to severe neurological disorders, like Alzheimer’s disease (AD) [2–4], there remains a pressing need to understand the extent of central molecular changes produced by obesity, and any sex differences that exist. Obesity exerts detrimental effects on the brain, where the hippocampus is particularly susceptible [5]. We previously reported that long-term obesity changed hippocampal methylation and associated gene expression [6]. Obesity decreases brain derived neurotrophic factor and impairs synaptic plasticity [6, 7], promotes neuroinflammation [8], impairs neurogenesis [7] and CA1 long-term potentiation [9], alters AMPA and NMDA receptor signaling [10, 11], and impairs learning across a variety of hippocampal-dependent rodent tasks and humans [12]. Unfortunately, females have been poorly represented across these studies. Prior to menopause, women have a lesser degree of adiposity and accompanying metabolic syndrome (lower rates of Type 2 Diabetes, non-alcoholic fatty liver disease, fasting glucose, and bad cholesterol, etc.) than men, but post-menopause, women’s weight and risk of metabolic-linked diseases generally increases [13]. Although limited work has been done, animal models generally support these findings. Young female mice demonstrated higher baseline levels of neurogenesis compared to males; four months of obesity reduced neurogenesis in females to levels commensurate with males and induced greater microglial activation [14]. Pathway analysis of hippocampal samples from female non-human primates on a long-term obesogenic diet indicate enrichment of neuroinflammatory pathways [15]. Overwhelmingly, studies support negative changes to brain systems and learning with obesity; females are grossly underrepresented in these studies and the extent of molecular changes due to obesity on vulnerable brain regions, like the hippocampus, remain poorly understood in them.

Autophagic and lysosomal pathway (ALP) activity is crucial in non-mitotic cells, like neurons, that do not have other means of diluting cellular waste accumulation that can lead to apoptosis. Studies support a role for ALP in memory formation and synaptogenesis [16] and has been linked to most types of neurodegenerative disease [17]. Despite the invaluable role ALP plays in neuronal homeostasis, sex differences in ALP or in response to obesity have not been characterized. Data from hypothalamic studies indicate a strong connection between obesity and downregulation of ALP [18–20], where females also show some resistance to inflammatory and autophagic changes [21]. Methods to enhance ALP, including caloric restriction (CR) [22, 23], improve functional outcomes in the hippocampus [24–29]. Due to the reliable increase in neurological risk conferred by obesity, the lack of female representation, and dearth of studies examining ALP in the hippocampus among these variables, we sought to fill gaps in the literature by examining interactions between sex and obesity. Here, we placed female and male C57BL/6 mice on a long-term obesogenic diet, and then placed half the mice on CR, for the purpose of measuring signaling and gene changes in the hippocampus associated with ALP. We also measured cognition with the Morris watermaze. We hypothesized that obesity would severely perturb ALP markers in the hippocampus, CR would restore some levels, and female and male mice would diverge in which signaling and gene markers were changed by dietary manipulation.

## Results

### Females and males differ in body weight and glucose tolerance

**Fig. 1:**
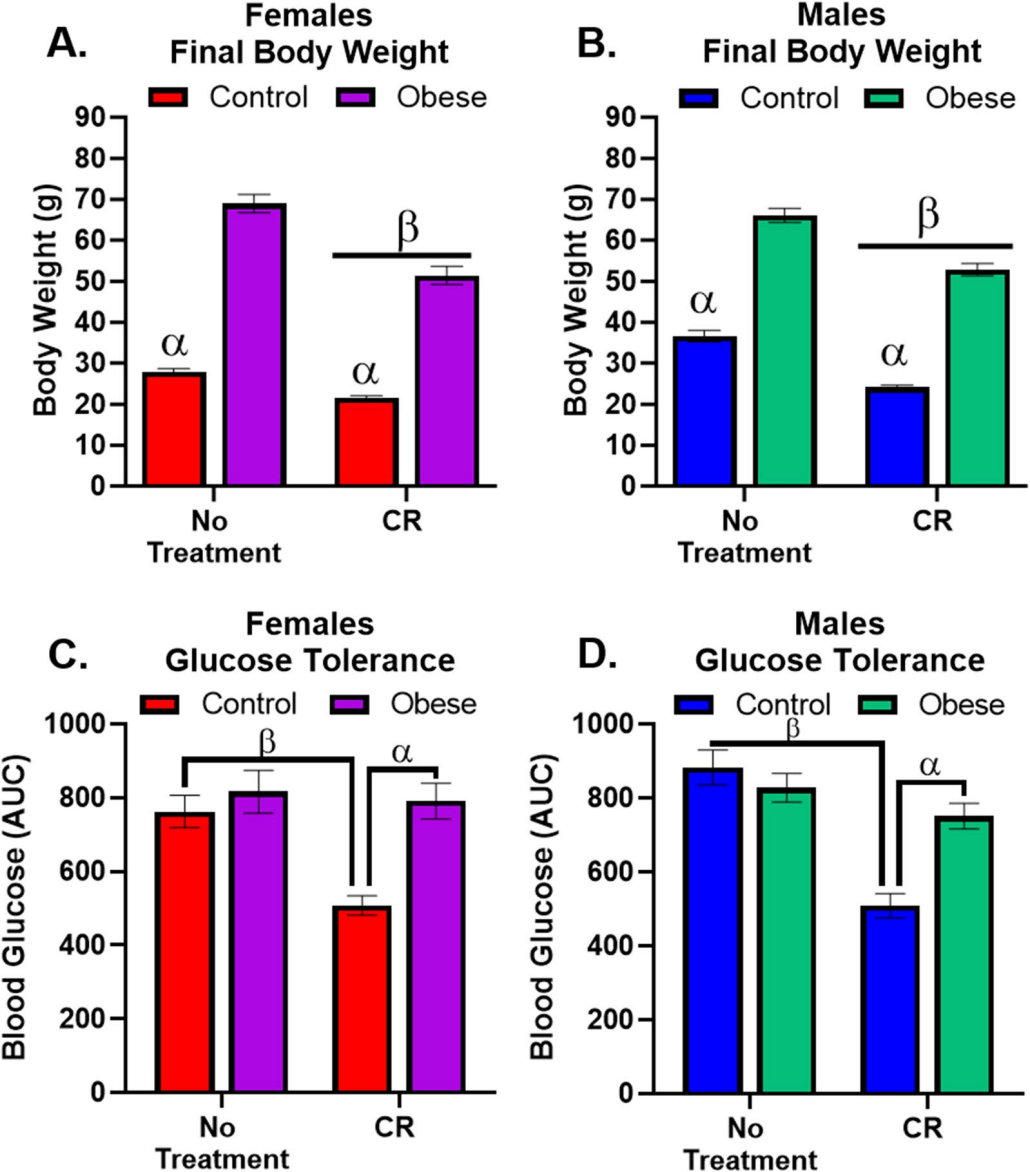
Peripheral Measures. **A** There was a significant interaction (F_1,32_ = 14.21, p = 0.0007) whereby Obese females gained significantly more weight than Control females. CR reduced body weight regardless of diet. **B** In males, there were significant main effects for diet (F_1,34_ = 486.5, p < 0.0001) and treatment (F_1,34_ = 95.8, p < 0.0001) where Obesity increased body weight, an effect attenuated by CR. **C** There was a significant interaction (F_1,32_ = 6.8, p = 0.014) in female fasting glucose. CR reduced fasting glucose in Control females only. **D** There was a significant interaction (F_1,34_ = 14.7, p = 0.0005). CR reduced fasting glucose in Control males only. α indicates significant effect due to diet (Control vs. Obese), β indicates significant effect due to treatment (No treatment vs. CR). All significance indicators represent a p-value of less than 0.05 unless otherwise indicated

Both females and males on an obesogenic diet gained significantly more weight than their Control counterparts (Fig. 1A, B), but with differing longitudinal patterns (Additional file 1: Fig. S1). CR reduced body weight in both Obese and Control females and males. Surprisingly, Obesity did not change glucose tolerance in females or males; however, CR significantly improved glucose tolerance in female and male Control mice only (Fig. 1C, D).

### Female cognition was more affected by obesity and CR

**Fig. 2:**
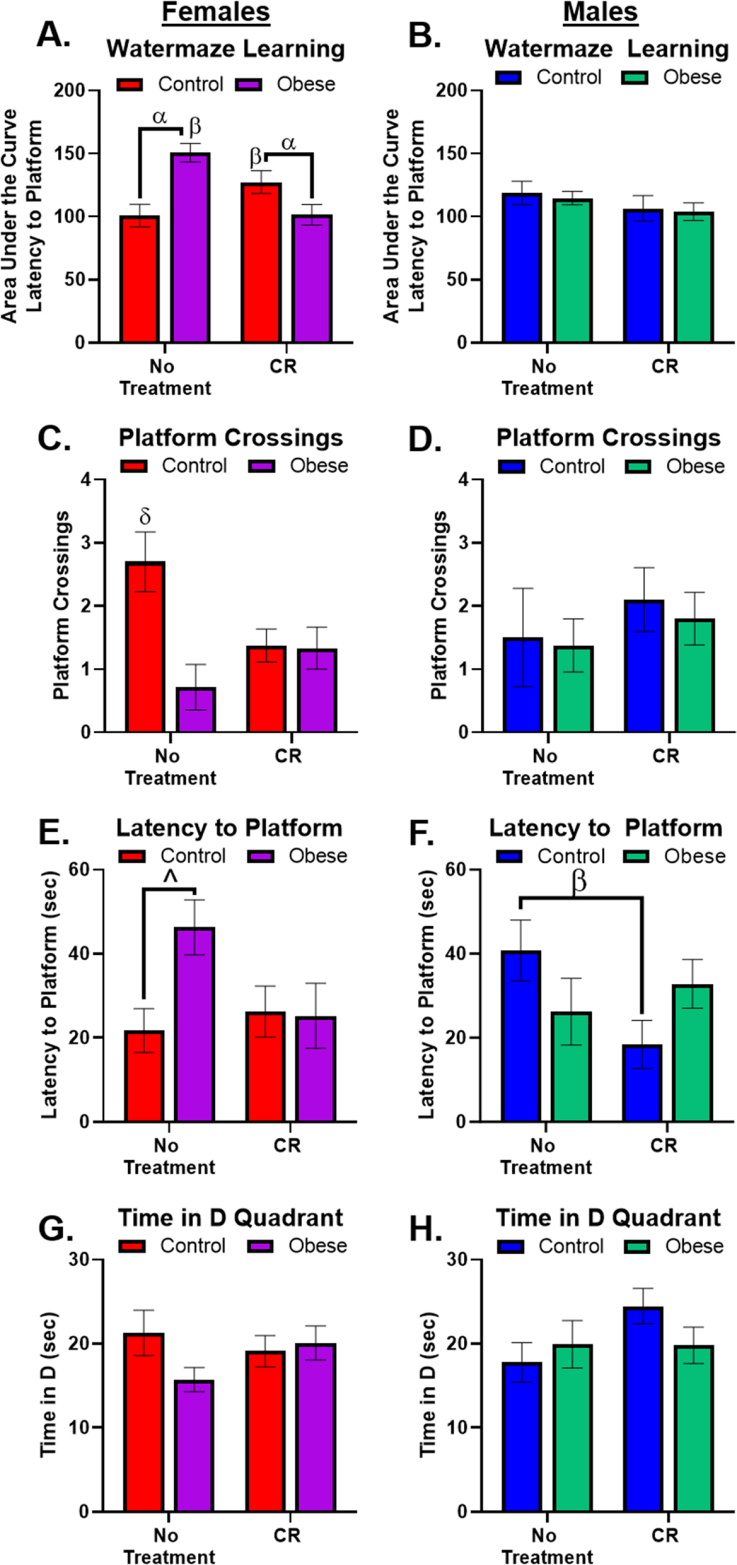
Watermaze Learning. **A** and **B** Learning across four days of training was expressed as area under the curve; the higher the value the poorer the learning. **A** There was a significant interaction for female learning (F_1,30_ = 18.9, p = 0.0001). Obesity produced learning deficits, but CR improved learning in Obese, but impaired learning in Control females. **B** There were no significant effects of diet or treatment in male learning behaviors. **C** There was a significant interaction (F_1,30_ = 6.3, p = 0.02) in platform crossings, Obese mice made fewer crossings, while CR reduced crossings for Control females. **D** There were no significant effects of diet or treatment in male platform crossings. **E** There was a trending interaction (F_1,30_ = 3.8, p = 0.06) and main effect of diet (F_1,30_ = 3.3, p = 0.08), significant post hoc tests confirm that Obesity alone produced increased latencies to finding the platform location. **F** There was a significant interaction (F_1,34_ = 4.7, p = 0.04) where CR in Chow males only reduced latencies to find the platform location. **G** There were no effects of diet or treatment in females on time in D quadrant (where the platform was located). **H** There were no effects of diet or treatment in male mice on time in D quadrant (where the platform was located). α indicates sig. difference due to diet (Control vs. Obese), β indicates sig. difference due to treatment (No treatment vs CR), δ indicates sig different from all other groups, ^ indicates strong trend (0.09 ≥ p ≥ 0.05) in ANOVA results with significant post hoc test. All significance indicators represent a p-value of less than 0.05 unless otherwise indicated

Learning behavior, measured by Morris watermaze, is a validated task for measuring hippocampal-dependent function [30] and is sensitive to perturbation from high-fat diets [31]. Overall, female cognition was more affected by diet and treatment conditions than the males’ behavior. Obesity reduced learning (Fig. 2A, Additional file 2: Fig. S2A), and probe retention measures (Fig. 2C, E) in females; a similar effect was observed in Time in D Quadrant (where the platform was located; Fig. 2G), but was not significant. CR rescued learning and latency to platform in Obese female mice, while impairing learning and probe measures in the Control females. There were no changes in male learning (Fig. 2B, Additional file 2: Fig. S2B) or any probe measures due to Obesity (Fig. 2D, F and H). Male Control mice did benefit from CR in one probe measure, latency to platform (Fig. 2F), an effect not observed in Obese mice. This effect was mirrored in Time in D Quadrant (where the platform was located) probe measure (Fig. 2H); however, the ANOVA effects were not significant. Overall, Obesity negatively impacted female learning and probe retention measures, while CR improved metrics in Obese females and slightly in control males, while impairing performance in Control females.

### Males, but not females, show expected changes to ALP phosphorylation pathways

**Fig. 3:**
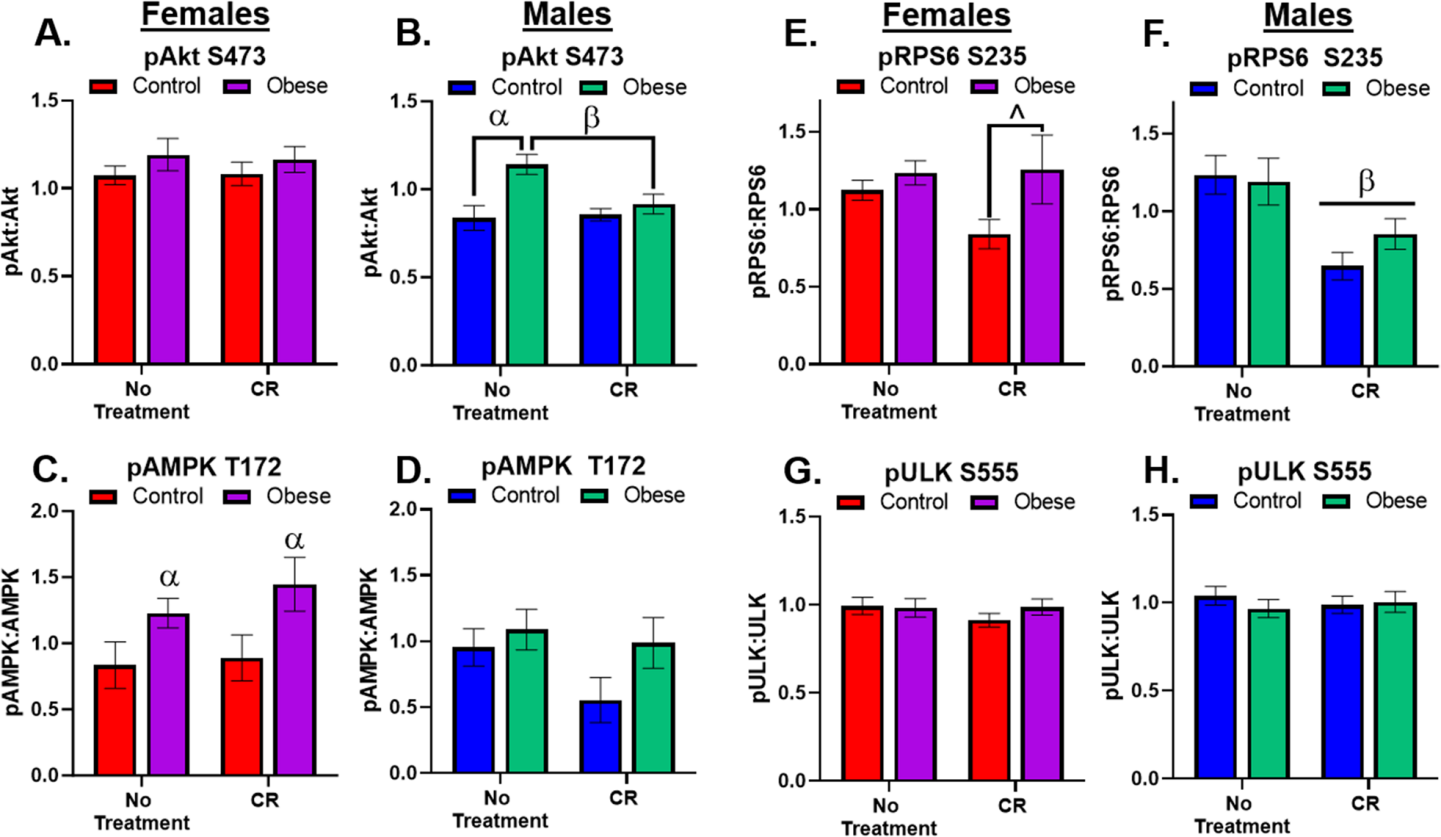
Signaling Pathways. **A** There were no effects of diet or treatment in female mice on pAkt expression. **B** There was a significant interaction (F_1,33_ = 4.8, p = 0.04) in males whereby Obesity increased pAkt levels compared to Control, while CR reduced pAkt levels in Obese to levels commensurate with Control mice. **C** There was a significant main effect of diet (F_1,30_ = 6.7, p = 0.01), pAMPK levels were elevated in Obese females, significantly so in the CR treatment group. **D** There were no effects of either diet or treatment on pAMPK levels in males **E**. There was a strong trend (F_1,29_ = 3.5, p = 0.07) in females; within CR treatment Obese females had increased levels of pRPS6 compared to Control females. **F** There was a significant main effect of treatment (F_1,32_ = 16.1, p = 0.0003) in male pRPS6 expression. CR reduced pRPS6 regardless of diet. **G** There were no effects of diet or treatment in pULK in female mice. **H** There were no effects of diet or treatment in pULK levels in male mice. α indicates significant effect due to diet (Control vs. Obese), β indicates significant effect due to treatment (No treatment vs. CR), ^ indicates strong trend (0.09 ≥ p ≥ 0.05) in ANOVA results with significant post hoc test. All significance indicators represent a p-value of less than 0.05 unless otherwise indicated

Lysosomal degradation heavily controlled by the mechanistic target of rapamycin (mTOR) [32]; furthermore, mTOR activity can be modulated by several other signaling factors including pAkt and pAMPK [33, 34]. As such, we measured several signal transduction proteins involved in mTOR modulation and signaling. Phosphorylation status of four proteins (Akt, AMPK, RPS6, and ULK1) related to initiation and maintenance of ALP were measured. Akt is a primary target of insulin signaling for triggering the uptake of glucose and can mediate mTOR signaling [35]. Females showed no changes across diet or treatments in pAkt levels (Fig. 3A). This could mean that Obese females developed hippocampal insulin resistance; however, given that CR had no effect to alter pAkt in either Control or Obese females, it seems more likely that pAkt was simply unaffected by either obesity or CR in females. In contrast, males demonstrated a dynamic response (Fig. 3B) of increased pAkt with Obesity, an effect attenuated by CR. This is an anticipated effect when surplus carbohydrate intake occurs and supports that obesity had not occurred to such a great extent that insulin resistance had occurred.

AMPK, which is activated by periods of energy deficits (exercise, fasting, etc. [36]) was surprisingly only increased by CR in Obese females (Fig. 3C), and had no effect in males (Fig. 3D). We used pRPS6 as a marker of mTOR activation [37]. There was a trending effect for CR to reduce pRPS6 in Control females only (Fig. 3E). CR was far more effective in males, where both Control and Obese groups had decreased pRPS6 (Fig. 3F). Results in males are commensurate with the established literature, but the lack of similar significant effects in females highlights that current studies may not translate or be representative of what occurs in female brains.

Finally, actions of ULK1 are dictated by where its phosphorylated. We focused on site Ser555, which is AMPK-dependent for increasing autophagy [38]. There were no differences in females (Fig. 3G) or males (Fig. 3H) due to diet or CR treatment in pULK levels.

### Males show greater changes to autophagy and amino acid sensing gene transcripts

**Fig. 4:**
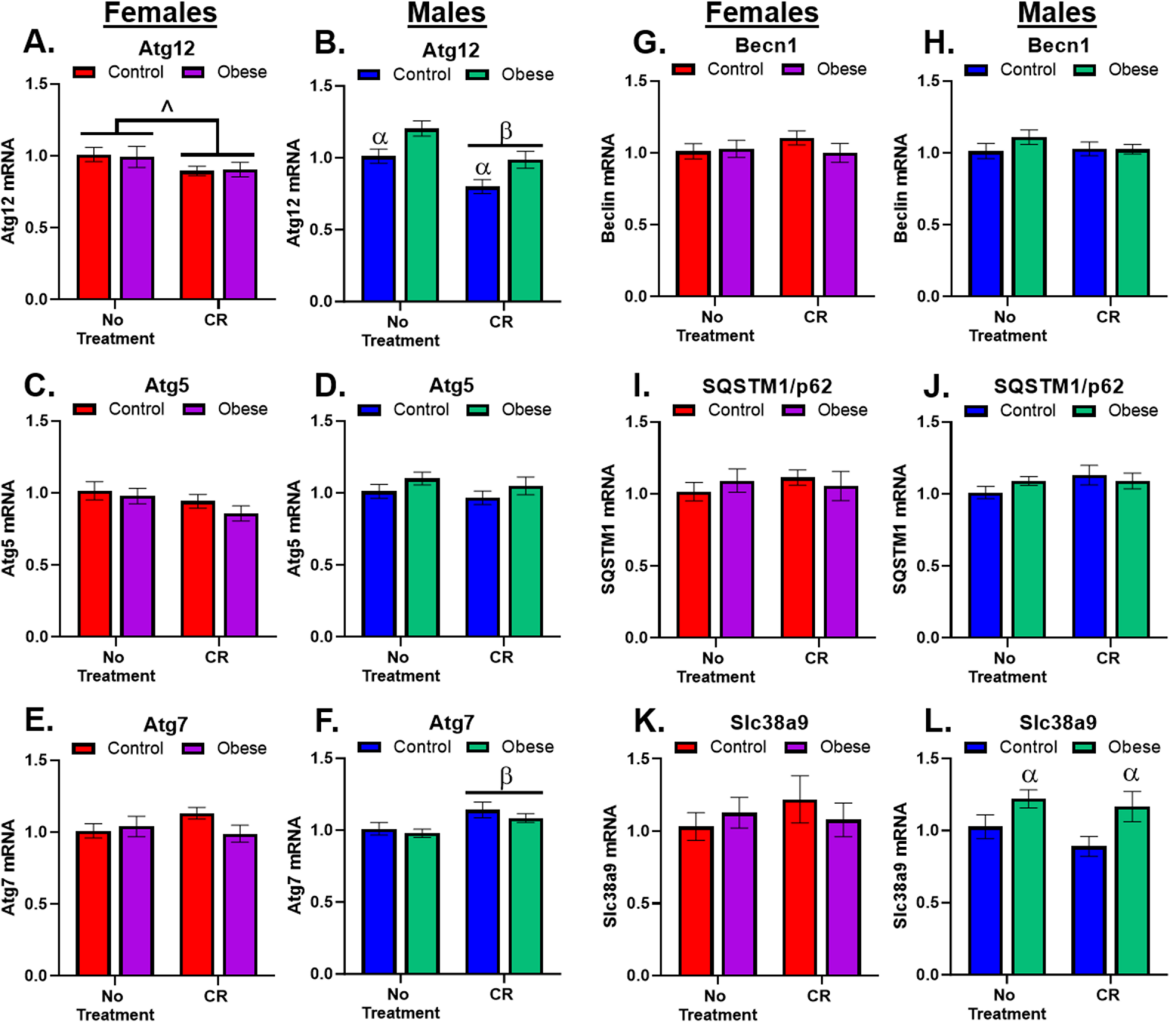
Autophagy Genes. **A** There was a strong trend for a treatment main effect (F_1,31_ = 4.1, p = 0.05) where CR reduced *Atg12* transcripts, regardless of diet. **B** There were significant main effects of both diet (F_1,33_ = 12.7, p = 0.001) and treatment (F_2,33_ = 16.3, p = 0.0003, whereby Obesity increased *Atg12* transcripts across treatment groups, but CR reduced *Atg12* in both Chow and Obese mice. **C** There were no effects of diet or treatment on *Atg5* transcripts in female mice. **D** There were no effects of diet or treatment on *Atg5* transcripts in male mice. **E** There were no effects of diet or treatment on *Atg7* transcripts in female mice. **F** There was s significant main effect of treatment (F_1,33_ = 7.5, p = 0.01), CR increased *Atg7*, particularly in Chow males. **G** There were no effects of diet or treatment on *Becn1* in female mice. **H** There were no effects of diet or treatment on Beclin1 in male mice. **I** There were no effects of diet or treatment on *SQSTM1* in female mice. **J** There were no effects of diet or treatment on *SQSTM1* in males. **K** There were no effects of diet or treatment in females on *Slc38a9*. **L** There was a significant main effect of diet (F_1,33_ = 8.1, p = 0.008), Obesity elevated *Slc38a9* levels, particularly in CR males. α indicates significant effect due to diet (Control vs. Obese), β indicates significant effect due to treatment (No treatment vs. CR), ^ indicates strong trend (0.09 ≥ p ≥ 0.05) in ANOVA results with significant post hoc test. All significance indicators represent a p-value of less than 0.05 unless otherwise indicated

We examined several autophagy-related genes, whereby alternations in mRNA expression can be indicative of functional changes in autophagy. Autophagy-related genes (Atg’s) 12, 5, and 7 are involved in the formation of the autophagosome from the phagophore [39], while *Atg5* and *Atg12* were previously found to be elevated in AD [40]. *Atg12* transcripts *were* consistently downregulated by CR; in females (Fig. 4A) the effect was modest, while in males, Obesity significantly elevated *Atg12* mRNA, while CR attenuated levels in both diet groups (Fig. 4B). There were no changes in *Atg5* mRNA in females (Fig. 4C) or males (Fig. 4D). *Atg7* mRNA was unaffected in females (Fig. 4E), but was increased by CR in Control males only (Fig. 4F). Beclin1 (Becn1) is a downstream target of ULK1 [41]. As there were no significant changes in ULK1 phosphorylation, not surprisingly, there were no significant effects of diet or CR treatment in females (Fig. 4G) or males (Fig. 4H) on *Becn1* transcripts. Sequestisome 1 (SQSTM1) is involved in the recognition of ubiquitin for identification of waste requiring sequestering by the phagophore [42]. There were no effects of diet or CR treatment on *SQSTM1* mRNA in females (Fig. 4I) or males (Fig. 4J). Solute carrier 38 a9 (slc38a9) is an amino acid sensor required for arginine activation of mTORC1 [43]. *Slc38a9* mRNA was unchanged in female mice (Fig. 4K), but was significantly increased by Obesity in male mice (Fig. 4L).

### Females show greater changes in transcripts related to lysosomal activation and degradation

**Fig. 5:**
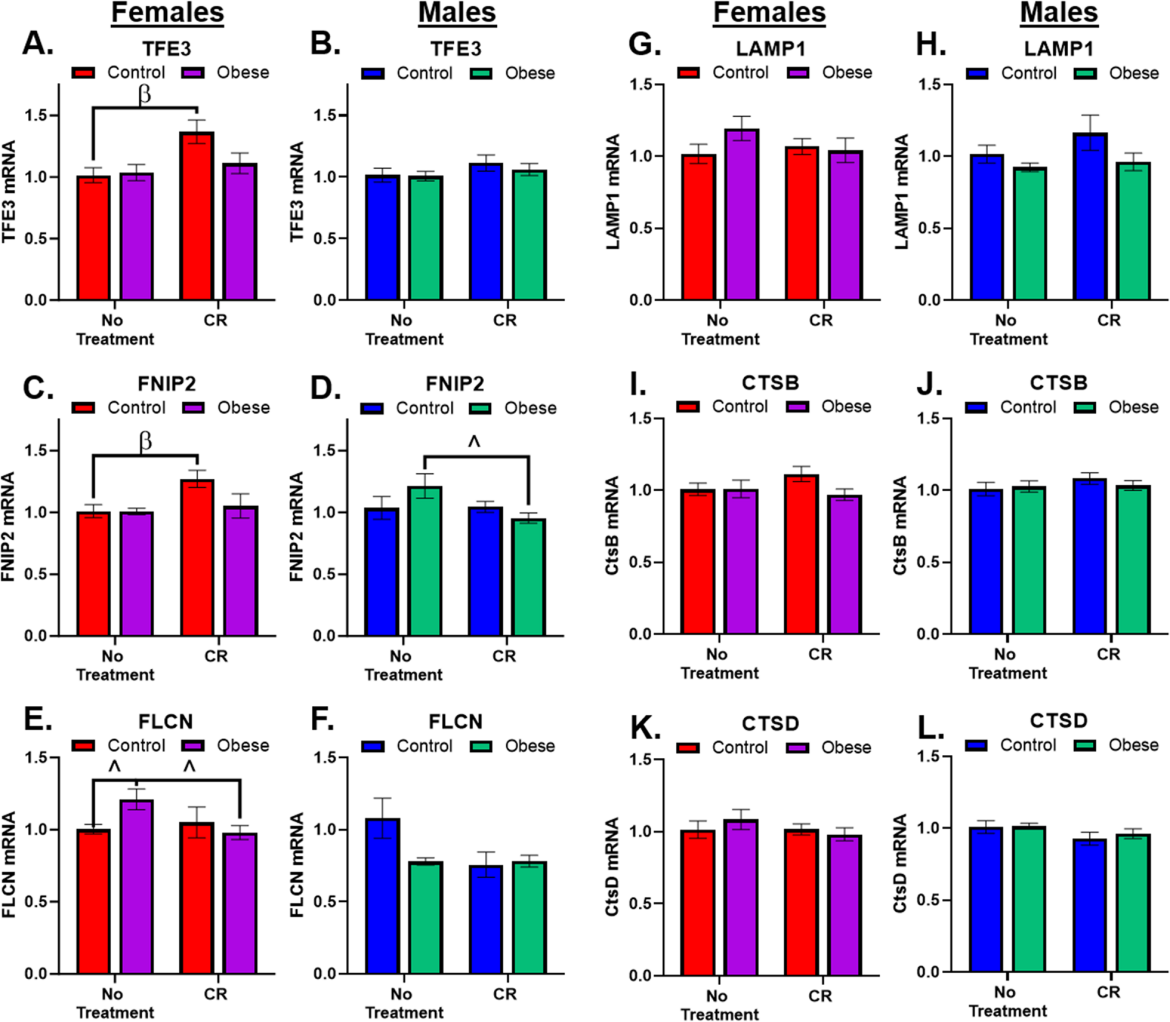
Lysosomal Genes. **A** There was a significant main effect for treatment (F_1,31_ = 6.9, p = 0.01), CR increased *TFE3* compared to no treatment, in Chow mice only. **B** There were no significant effects on *TFE3* in males. **C** There was a significant main effect for treatment (F_1,31_ = 4.6, p = 0.04), CR increased *FNIP2* in Chow females only. **D** There was a strong trending interaction (F_1,33_ = 3.3, p = 0.08), CR reduced *FNIP2* transcripts in Obese males only. **E** There was a strong trending interaction (F_1,29_ = 3.6, p = 0.07) where higher levels of *FLCN* in Obese females were reduced by CR. **F** There were no significant effects of diet or treatment on *FLCN* levels in male mice. **G** There were no significant effects of diet or treatment on *LAMP1* in female mice. **H** There were no significant effects of diet or treatment on *LAMP1* levels in male mice. **I** There were no effects of diet or treatment on *CTSB* in female mice. **J** There were no effects of diet or treatment in males on *CTSB*. **K** There were no effects of diet or treatment in females on *CTSD*. **L** There were no significant effects of diet or treatment on *CTSD* in male mice. α indicates significant effect due to diet (Control vs. Obese), β indicates significant effect due to treatment (No treatment vs. CR), ^ indicates strong trend (0.09 ≥ p ≥ 0.05) in ANOVA results with significant post hoc test. All significance indicators represent a p-value of less than 0.05 unless otherwise indicated

We evaluated lysosomal activity via measuring mRNA transcripts of several genes where altered levels may be indicative of changes in activity, including several genes identified in the coordinated lysosomal expression and regulation (CLEAR) network (FNIP2, LAMP1, CTSB, CTSD) [44]. Transcription factor E3 (TFE3), like transcription factor EB (TFEB), is a master regulator of ALP genes [45], but unlike TFEB, it was identified as being significantly upregulated in early Braak Stage III patients [40] in a hypothesized attempt to upregulate ALP to cope with increasing neurotoxic pathology [40]. Surprisingly, *TFE3* mRNA was only changed in female Control mice following CR (Fig. 5A), males were unaffected (Fig. 5B).

Folliculin (FLCN) is a GTPase activating protein that modulates lysosomal activity in response to nutrient availability [46], it also directly interacts with AMPK, TFEB, and TFE3 [47], but its role in the brain has yet to be studied. Its actions are directed by its interacting proteins, FNIP1 and FNIP2. Interestingly, in females, effects on *FNIP2* mRNA (Fig. 5C) were identical to *TFE3* (Fig. 5A), suggesting in the brain these proteins may be linked. There was also a trending effect for CR to reduce *FNIP2* mRNA in Obese males (Fig. 5D); the effect in males did not resemble effects of *TFE3*. *FLCN* was also only changed in females, albeit as a trending effect, for moderately increased levels of *FLCN* in Obese mice that were attenuated by CR (Fig. 5E). There were no significant effects of diet or CR treatment on *FLCN* in male mice (Fig. 5F).

Lysosomal associated membrane protein-1 (LAMP1) is a glycoprotein component of the lysosomal limiting membrane [48]. There were no significant changes in *LAMP1* mRNA in females (Fig. 5G) or males (Fig. 5H). Cathepsins, like B (CTSB) and D (CTSD) are hydrolases responsible for degrading lysosomal contents, and both have been linked to AD [40, 49]. There were no changes due to diet or CR treatment in *CTSB* (Fig. 5I, J) or *CTSD* mRNA (Fig. 5K, L).

## Discussion

The purpose of this study was to determine whether obesity and/or CR affected key ALP signaling and genetic markers in the hippocampus of both female and male mice. In line with our hypothesis, obesity affected ALP measures in sex-specific ways; females showed changes mostly to lysosomal genes, while males had more robust changes in autophagy genes and signaling pathways. Neither females nor males had a peripheral or central indicator of insulin resistance, so the effects observed were specific to obesity. Previous studies have focused on insulin resistance as a driving factor in cognitive decline and eventual neurodegeneration associated with obesity [50]; however, in neither females nor males, did peripheral glucose control or central measures of insulin signaling align with cognitive performance. These reports support the need to understand how obesity perturbs major molecular functions in the hippocampus in sex-specific ways, that precede development of Type 2 Diabetes, to increase susceptibility to neurological dysfunction.

**Fig. 6:**
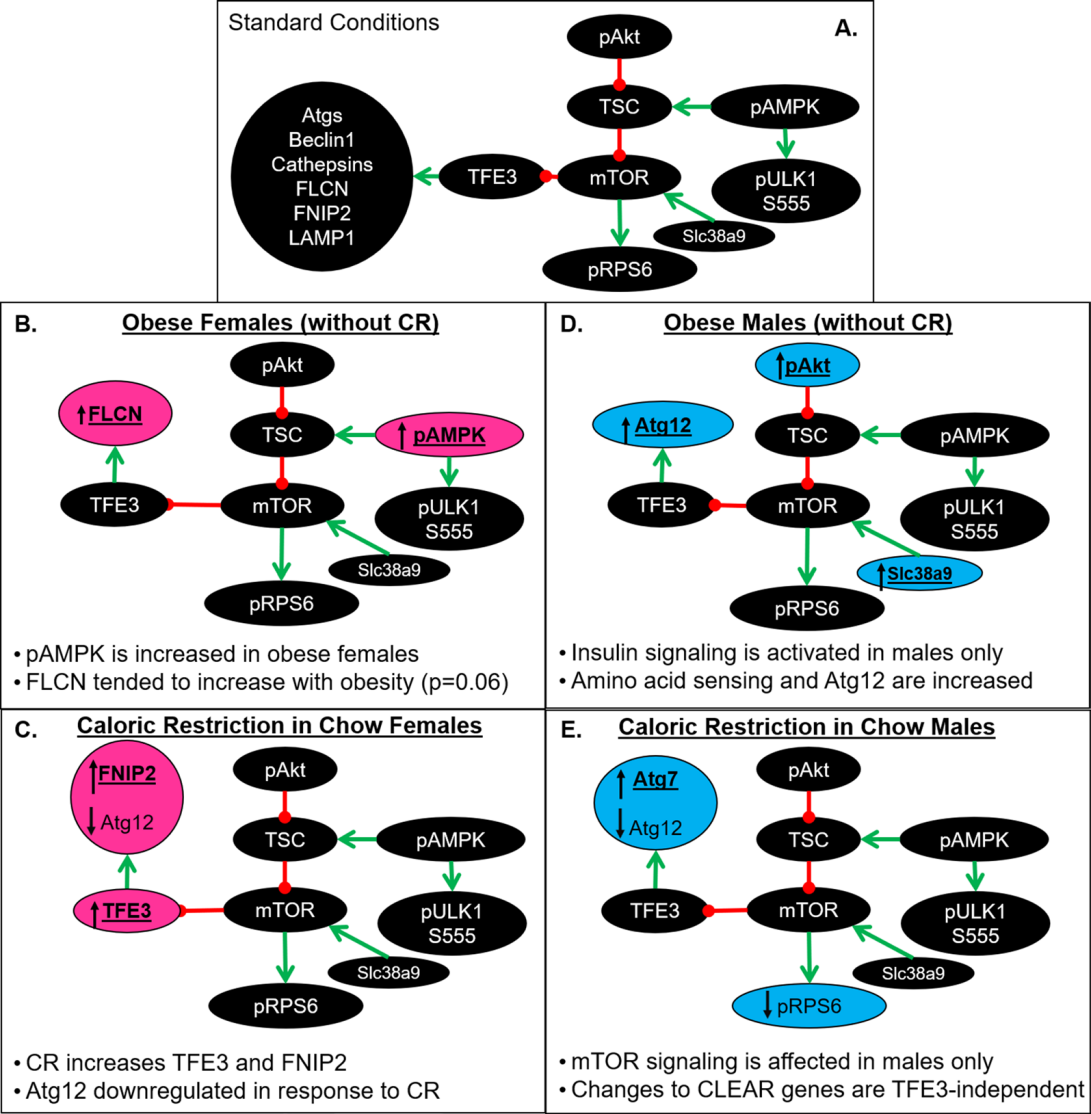
Depicts sex-specific changes due to obesity and CR. **A** illustrates the connections between the ALP signaling and genomic pathways measured in this study. **B** and **C** Females were more resistant to metabolic and genomic changes due to obesity than males. In males, expected results were observed via changes in pAKT and mTOR signaling. **D** and **E** CR differentially affected females and males; TFE3 was affected in females only and may relate to the genomic changes observed in FNIP2 and Atg12; however, in males Atg7 and Atg12 effects may be via a different transcription factor, like TFEB, which is known to be tied to mTOR signaling

Females showed cognitive impairment with obesity, and while CR was beneficial in reversing the negative effects of obesity on 4 day learning in Obese females, it was detrimental to Control females. Late application of CR to females was previously associated with reduced watermaze performance [51], indicating that extreme weight loss, or being underweight, is more detrimental to mid-aged to old females than males. Although several molecular ALP markers changed in female mice due to CR (Fig. 6), none of them aligned with cognitive performance, suggesting factors outside of those measured, likely via NMDAR functioning [52, 53] are driving learning effects. This paradigm of obesity increased pAMPK levels in females, while Obesity prevented the expected reduction of pRPS6 by CR, indicating Obese female mice were not reaping the benefits of suppressed mTOR signaling attributed to CR. Generally, autophagic markers were unchanged in females across conditions, with the exception of a trending decrease in *Atg12* mRNA levels due to CR, which should be interpreted cautiously, but is worth noting that this was the only similarity observed between females and males (Fig. 6).

In contrast to autophagy genes, lysosomal transcripts were dynamically regulated by obesity and CR in females. Interestingly, *TFE3* and *FNIP2* co-varied with CR in tandem. CR increased transcripts of both *TFE3* and *FNIP2* in Control females only, while Obesity prevented the CR-induced increases. The opposite effect was observed in *FLCN* transcripts. Obesity elevated *FLCN* mRNA, an effect attenuated by CR. CR-mimetics have previously been found to increase TFE3 activity [54, 55], but no link has previously been made between CR and any component of the folliculin-complex. FLCN is a RagC/D GTPase-activating protein, that binds with FNIP1 and FNIP2 to form a complex that regulates mTORC1 activity to modulate lysosomal biogenesis and autophagy, and may also directly interact with AMPK [56]. Substantial evidence links FLCN, FNIP2 and TFE3; however, these effects vary greatly based on tissue type, and neither have, currently, been characterized in the brain. Manipulation of FLCN and FNIP2 active sites prevents GAP activity leading to nuclear retention of TFE3 [57]. In cancer tissues, loss of FLCN leads to greater TFE3 activation and induction of related pathways [58]. Although, presently, a direct relationship between FLCN and/or FNIP2 with TFE3 cannot be determined from these results, requiring direct manipulations within neural tissue, it is suggestive of greater dependency between these two proteins, in females; as the *TFE3* gene is on the X chromosome, this may explain the absence of a corresponding effect in males. FInally, the increase in TFE3 mRNA is not necessarily indicative of increased TFE3 activity; reliable antibodies for phosphorylated TFE3 in mouse were not available to us for this study, but would provide valuable information for future studies for how TFE3 behaves due to obesity and sex.

Surprisingly, there were no changes to *LAMP1*, *CTSB*, or *CTSD* across conditions in females or males. Examining mRNA for these genes is a basic measure of coordinated lysosomal expression and regulation (CLEAR) element, as all three are genomic targets of TFEB, and can signal overall upregulation of the CLEAR network of genes that augment autophagic/lysosomal activity [44]. Transcript levels, however, do not inform on lysosomal distribution or functionality, which would require IHC co-labeling of LAMP1 with hydrolases (like CTSB and CTSD) [48]. More targeted evaluation of these targets may reveal different results, and provide insight into whether obesity affects distribution, trafficking, and/or co-localization of lysosomes throughout neurons in the hippocampus, in lieu of null effects on mRNA expression.

The lack of behavioral change in males was surprising, but not unprecedented. Leptin resistant male mice, although twice the size of control mice, had similar performance in watermaze [59], while 16 or 24 weeks of obesity had no effect across watermaze learning days in male C57BL/6 mice [60, 61]. Aside from nuances in watermaze difficulty, duration of dietary exposure may affect the cognitive impact of obesity. Most obesity studies keep rodents on diets for 3–6 months, our protocol was designed to be more clinically relevant and representative of a population that is increasingly becoming overweight young and remaining obese into middle age. Females were at first resistant to developing obesity (Additional file 1: Fig. S1), but after a year on the diet eventually succumb to the negative effects of the diet. Males however, are quick to become obese and may, over such a long duration, adapt and compensate for the prolonged state of obesity.

Although they performed comparably in task learning, severe molecular changes still occurred within the hippocampus of males. As expected, pAkt was increased in Obese males only, and attenuated by CR, while pRPS6 was suppressed by CR. Effects on autophagy genes were much more nuanced and unexpected. Atg12 is involved in autophagosome formation and is a target of TFEB and FOXO transcription factors [62, 63]. *Atg12* mRNA was significantly upregulated in Obese males, regardless of CR treatment, but also ubiquitously decreased by CR, albeit only mildly in females. This effect was surprising given that loss of Atg12 has been found to reduce the lifespan of *C. Elegans* [64]. Although CR is associated with upregulating autophagy and extending lifespan, the analysis of *Atg12* transcripts in the brain following long-term CR is surprisingly absent from the literature. At best, we can speculate that since Obesity increased *Atg12* levels in males, and since upregulation of Atg12 has been observed in CA1 from AD patients [40], that cellular upregulation of Atg12 represents an early response to inflammation/cellular dysfunction as a means of upregulating ALP for coping with cellular distress; and conversely, the decrease in *Atg12* mRNA due to CR may be representative of improved cellular efficiency. A similar effect of obesity was detected in mRNA for *Slc38a9*, an amino acid (arginine) sensor on lysosomes which aides in mTORC1 regulation [57], and disassembly of the lysosomal-folliculin complex allowing FLCN GAP activation of RagC [65]. CR also increased levels of *Atg7*, but only in Control-fed mice. Atg7 is vital for autophagy induction, it drives the initial phagophore formation; Obesity prevented CR-induced increase in *Atg7* suggesting obesity interferes with all aspects of autophagy, making induction more difficult, while enhancing other aspects. Lysosomal changes in males were surprisingly absent, with the exception of a trending decrease in *FNIP2* mRNA in Obese males with CR, further highlighting the discrepancies between female and male responses to obesity and CR.

## Conclusions

The etiological cause of sporadic Alzheimer’s Disease remains unknown. Current clinical treatments focus solely on amyloid beta removal, the results of which are underwhelming and carry significant side effects [66, 67]. Rather than continuing to fixate on a histopathological marker under increasing scrutiny and skepticism, we should characterize cellular changes in experimental groups known to be at greater risk of developing AD and during the time period associated with disease initiation. Obesity and female sex are two of the highest risk groups for developing Alzheimer’s disease. Although the hippocampus is not the first brain region to show histopathological changes in AD, it is uniquely susceptible to the negative impacts of obesity, which can precipitate and exacerbate toxic accumulations, and perturb cognitive abilities. The role of the hippocampus in learning and memory also makes it ideal, over other brain regions, for measuring behavioral changes due to environmental manipulations. These results are, to our knowledge, the first to characterize sex differences and interactions with obesity in ALP at a critical age period, with results providing further support for the role of ALP in hippocampal-related dysfunction. An existing caveat is that we did not evaluate female mice following ovariectomy. Female hormonal loss and obesity are known to negatively impact Alzheimer’s disease outcomes in animal models [68, 69]. Menopause occurs during the predicted prodromal phase, marking the uncoupling of hormones from brain energy metabolism, resulting in a hypometabolic state [70] and, importantly, premenopausal women do not develop sporadic Alzheimer’s disease. The effects of either estrogen or progesterone, both potent neuroprotective hormones, on ALP is currently unknown, but will the subject of future studies.

## Materials and methods

All methods and procedures were approved by the Legacy Research Institute IACUC (protocol #123-20) prior to any rodent work occurring. All methods were performed in accordance with guidelines and regulations directed in the Guide for the Care and Use of Laboratory Animals and in full compliance with OLAW in an AAALAC accredited facility.

Mice were maintained on a 12/12 light cycle, lights on at 0730, and given ad libitum access to food and water (up to the 40-week treatment point when half were switched to CR).

### Mice

80 C57BL/6J female and male mice were purchased from Jackson Labs at 5 weeks of age. The n = 10/group size was chosen based on previous studies and behavioral outcomes and knowing that some mice on an obesogenic diet would not likely make it to the end of the study. Following a week of habituation, mice were randomly assigned by cage to receive standard Purina chow (Control) or a 60% high-fat diet (Research Diets). Following 40 weeks on their respective diets, mice were further randomly sub-divided into one of two treatment groups: No treatment or caloric restriction (CR). Caloric restriction methods were adopted from previous experiments [71] providing mice with a 40% CR, by weight, maintaining mice on their assigned control/obese diets. Mice were fed ~ 1700 daily, prior to lights off. CR treatments continued for 12 weeks. Mice were weight weekly for the duration of the project (Additional file 1: Fig. S1). Tissue collection occurred > 12h after CR mice received their last food allotment. Over the course of the 15-month study, seven mice on the obesogenic diet died (determined to be due to natural causes).

### Glucose tolerance test

A week prior to behavioral testing, mice received glucose tolerance testing (GTT). For GTT, mice were fasted for 6 h starting at 0800. A baseline blood glucose measurement was taken prior to mice receiving an i.p. injection of 1.5 g/kg of glucose in sterile PBS. Following bolus, blood glucose measurements were taken at 30, 60, 90, and 120 min post-injection using a glucometer (AgaMatrix Presto). Data was transformed into area under the curve (AUC) using GraphPad Prism.

### Morris watermaze

Watermaze testing took place 11 weeks after new dietary treatments were implemented. Experimenter was blind to conditions over the course of behavioral testing. The pool has a diameter of 122cm, was filled with ~ 21 °C water made opaque with non-toxic white tempera paint. The five-day protocol consisted of 4 days of training. Each day, mice were placed in each of four quadrants (A, B, C, D) in the pool and given 60 s to find the hidden platform, located in Quadrant D. If the mouse failed to find the platform, it was placed on the platform. Once on the platform, the mouse had 15 s to observe spatial cues. The order of quadrant placement was changed each day. On day five, the platform was removed and mice had 60 s to explore the pool. The latency to reach, the proximity to, and crossings over the location where the platform was were measured, along with the time spent in D quadrant (where the platform had been located). Learning across the four-day training was transformed into area under the curve using GraphPad Prism; the greater the area under the curve the poorer the learning. One female was excluded from the behavioral analysis due to an inability to swim properly.

### Tissue collection and processing

Hippocampus was collected from mice following anesthesia with isoflourane, followed by cervical dislocation and rapid decapitation. One hippocampi was reserved for western blot processing and the other for RNA isolation for qPCR analysis.

### Western blotting

Tissues were sonicated with RIPA buffer. Protein quantification with BCA assay and 40ug of protein loaded into each well. We used the BioRad XT criterion gel system with MOPS running buffer (#1610788). Proteins were transferred to PVDF membrane and blocked with 5% milk prior to antibody probing. We used the following primary antibodies: pAkt S473 (#8200S), Akt (#8200S), pAMPK T172 (#8208S), AMPK (#8208S), pRPS6 S235 (#8207S), RPS6 (#8207S), pULK S555 (#23988S), and ULK1 (#23988S), all were from Cell Signal and probed overnight at 4  C in TBST. Blots were incubated with secondary HRP antibody for 1 h prior to chemiluminescent treatment and visualization on our BioRad imager. Blots were analyzed with BioRad Image Lab software version 6.1. Raw western blot images are available as Additional file 3.

### qPCR

RNA isolation, preparation, and analysis was performed by the Shared Gene Profiling Core at Oregon Health & Science University (Portland, OR). In brief, RNA was isolated using Qiagen RNeasy mini kit with on-column DNase-treatment per manufacturers instructions and utilizing a QIAcube isolation robot. RNA quality was assessed and concentrations determined by Bioanalyzer. Reverse transcription was performed using SuperScript VILO cDNA synthesis kit (Invitrogen) using 126.5 ng of RNA. All samples were run in triplicate across 15 custom TaqMan Array Micro Fluidic Cards on QuantStudio 12K Flex Realtime PCR systems. Data was collected using Applied Biosystems QuantStudio 12K Flex Software v1.4. Taqman probes used included: *Atg12* (Mm00503201_m1), *Atg5* (Mm01187303_m1), *Atg7* (Mm00512209_m1), *Becn1* (Mm01265461_m1), *CTSB* (Mm01310506_m1), *CTSD* (Mm00515586_m1), *FLCN* (Mm00840973_m1), *FNIP2* (Mm01220192_m1), *LAMP1* (Mm00495262_m1), Slc38a9 (Mm00724649_m1), SQSTM1 (Mm00448091_m1), *TFE3* (Mm01341186_m1), with *GAPDH* (Mm99999915_g1) used as a housekeeping control. Two probes, *FLCN* and *LAMP1*, had several samples that returned with high standard deviations across triplicates, producing ∆∆Ct values that were outside two standard deviations from the mean. This resulted in six samples being removed from *FLCN* analysis and four being removed from *LAMP1*. These aberrations were not observed in other gene probes or the housekeeping gene, and were thusly attributed to inherent issues with the probes/cards themselves and not the samples.

### Experimental design and statistical analysis

Behavioral and biochemistry results were analyzed as 2-way ANOVAs with 2 (Control, Obese) × 2 intervention (no treatment, CR). Shapiro–Wilk tests for normality were performed and evaluated for each ANOVA. Statistical analysis was performed using GraphPad Prism v9. Planned comparisons used Fisher’s LSD test, while post hoc comparisons used Tukey corrections for multiple comparisons. Full p-values are provided for all statistical analyses, while the statistical indicators on the graphs depict only rejection of the null hypothesis, p-values < 0.05. Additionally, we report some trending main effects/interactions, which was defined as 0.09 ≥ p ≥ 0.05, and with a planned comparison that was significant, such as with *FNIP2* in males and *FLCN* in females. The graphical indicator for these effects is labeled as a “trend” and we emphasize in the results and discussion that these results should be interpreted cautiously. 

### Supplementary Information

**Additional file 1: Figure S1.** Depicts the longitudinal weight gain data collected from weekly weigh ins of the mice.

**Additional file 2: Figure S2.** Depicts daily watermaze learning in female and male mice that is depicted as AUC in Figure 2. Latency to reach the platform was measured each day across four trials for 4 days. There were no significant effects in the male mice. Female mice showed a significant diet x treatment interaction (F_1,30_=18.04, p=0.0002).

**Additional file 3. **Western blot images: All raw western blot images are provided. The phosphor/total antibody marker is labeled for each blot. Numbers at the top of each blot are subject numbers used in the represented data.

## Data Availability

The datasets generated during and/or analyzed during the current study are not publicly available, but are available from the corresponding author on reasonable request.
