# Supplementary figures and images for: Sex differences in response to obesity and caloric restriction on cognition and hippocampal measures of autophagic-lysosomal transcripts and signaling pathways
Source: BMC Neurosci. 2024 Jan 2;25:1. doi: 10.1186/s12868-023-00840-1 (PMC10759648; doi:10.1186/s12868-023-00840-1)

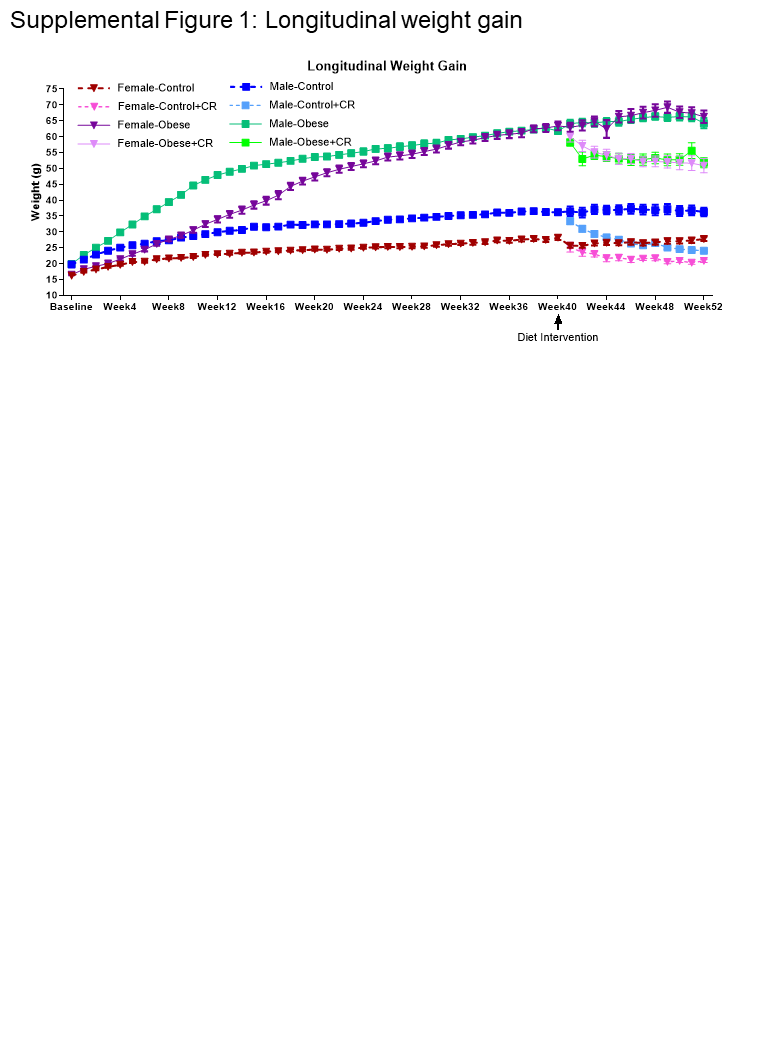

Supplement: Supplementary file 1 — Additional file 1: Figure S1. Depicts the longitudinal weight gain data collected from weekly weigh ins of the mice. [file 12868_2023_840_MOESM1_ESM.tif]

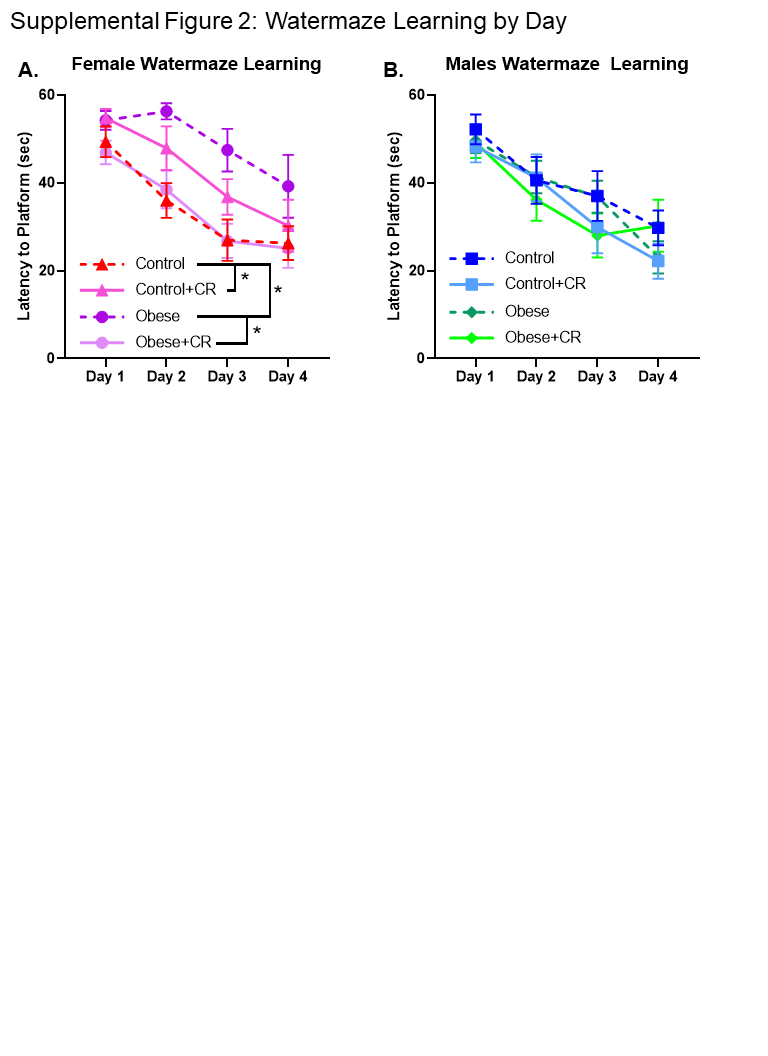

Supplement: Supplementary file 2 — Additional file 2: Figure S2. Depicts daily watermaze learning in female and male mice that is depicted as AUC in Figure 2. Latency to reach the platform was measured each day across four trials for 4 days. There were no significant effects in the male mice. Female mice showed a significant diet x treatment interaction (F1,30=18.04, p=0.0002). [file 12868_2023_840_MOESM2_ESM.tif]
