# Supplementary material for: Sex differences in response to obesity and caloric restriction on cognition and hippocampal measures of autophagic-lysosomal transcripts and signaling pathways
Source: BMC Neurosci. 2024 Jan 2;25:1. doi: 10.1186/s12868-023-00840-1 (PMC10759648; doi:10.1186/s12868-023-00840-1)
Supplement: Supplementary file 3 — Additional file 3. Western blot images: All raw western blot images are provided. The phosphor/total antibody marker is labeled for each blot. Numbers at the top of each blot are subject numbers used in the represented data. [file 12868_2023_840_MOESM3_ESM.pdf]

Blot 1A

Blot 1			
Well	Sub#	Sex	Condition
1	Ladder		
2	301	Male	Control
3	306	Male	Obese
4	312	Male	Chow+CR+CQ
5	320	Female	Obese+CR
6	321	Female	Control
7	326	Female	Obese
8	331	Male	Obese+CR
9	336	Male	Control+CR
10	341	Male	DIO+CR+CQ
11	351	Female	DIO+CR+CQ
12	356	Female	Control+CR
13	361	Male	Control+CR
14	366	Male	Obese+CR
15	371	Male	Control
16	378	Female	Obese
17	381	Female	Chow+CR+CQ
18	Ladder		

*Blacked out cells are samples from another project that were run alongside these samples, but are not represented in the results of this manuscript.

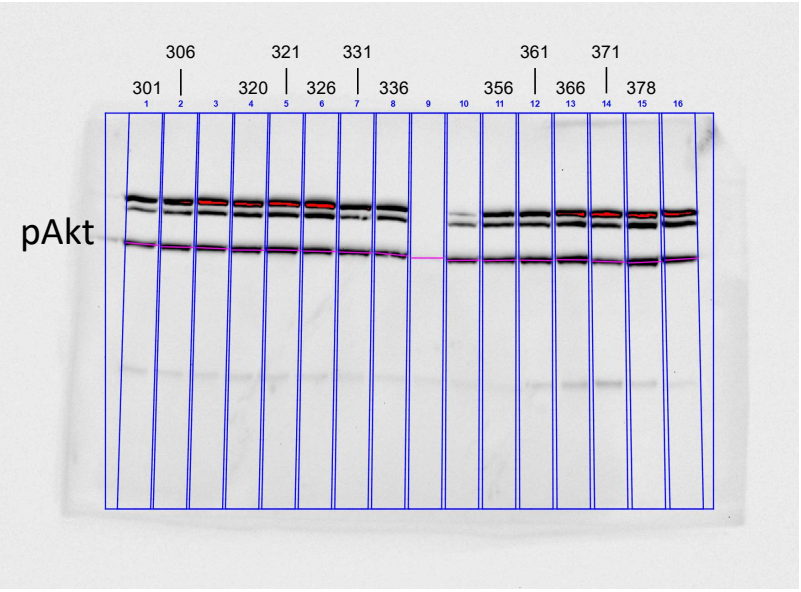

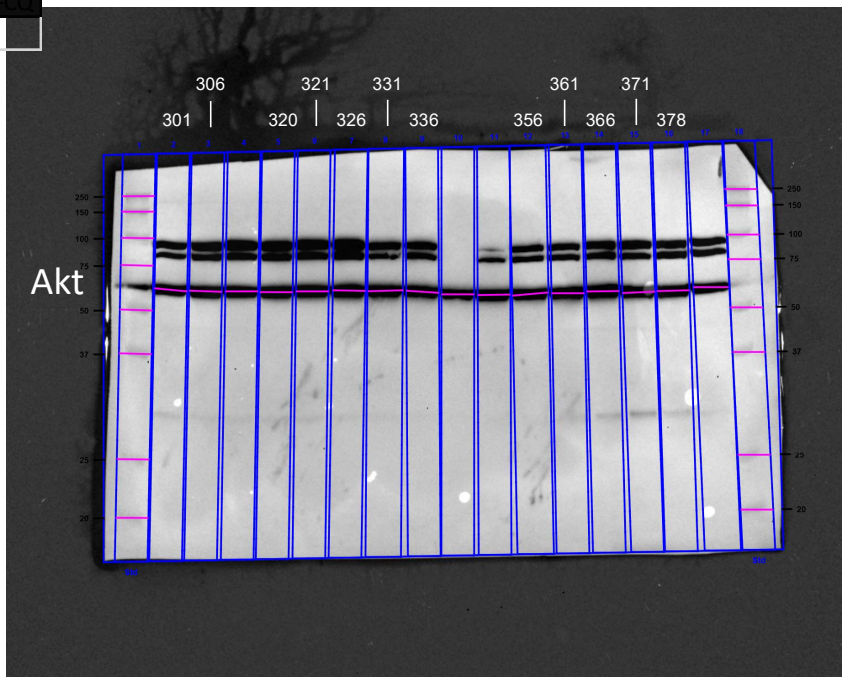

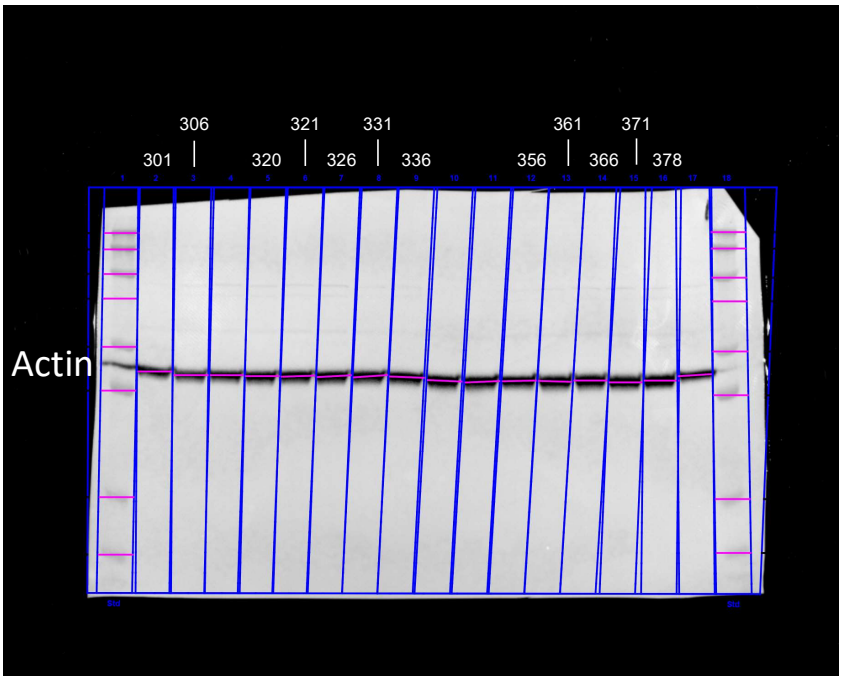

Blot 1B

Blot 1			
Well	Sub#	Sex	Condition
1	Ladder		
2	301	Male	Control
3	306	Male	Obese
4	312	Male	Chow+CR+CQ
5	320	Female	Obese+CR
6	321	Female	Control
7	326	Female	Obese
8	331	Male	Obese+CR
9	336	Male	Control+CR
10	341	Male	DIO+CR+CQ
11	351	Female	DIO+CR+CQ
12	356	Female	Control+CR
13	361	Male	Control+CR
14	366	Male	Obese+CR
15	371	Male	Control
16	378	Female	Obese
17	381	Female	Chow+CR+CQ
18	Ladder		

*Blacked out cells are samples from another project that were run alongside these samples, but are not represented in the results of this manuscript.

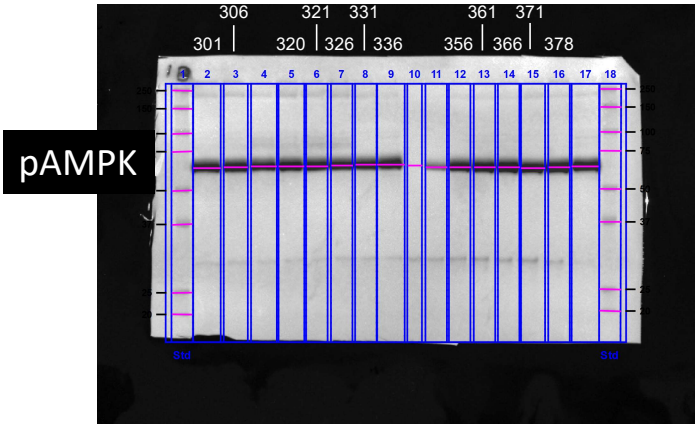

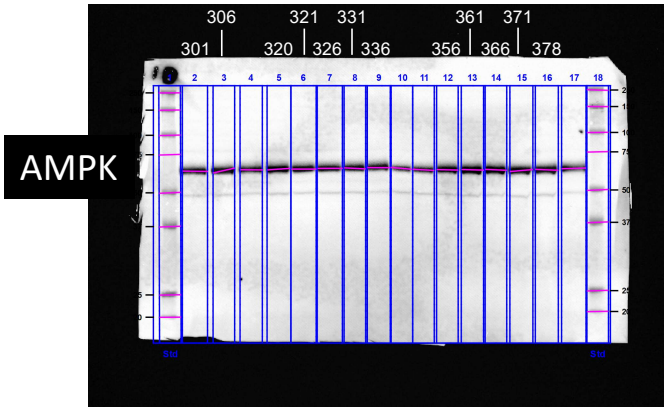

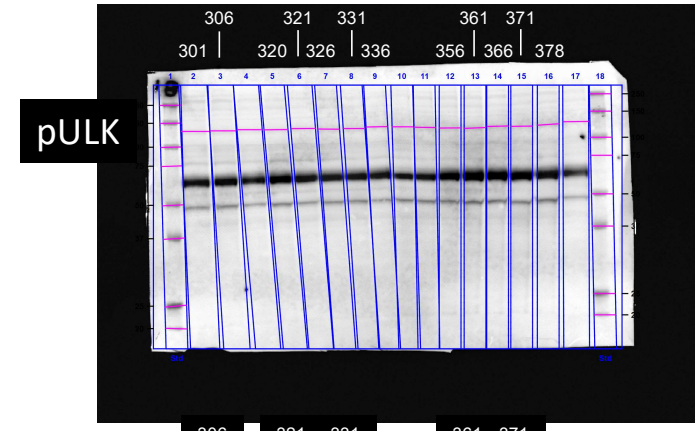

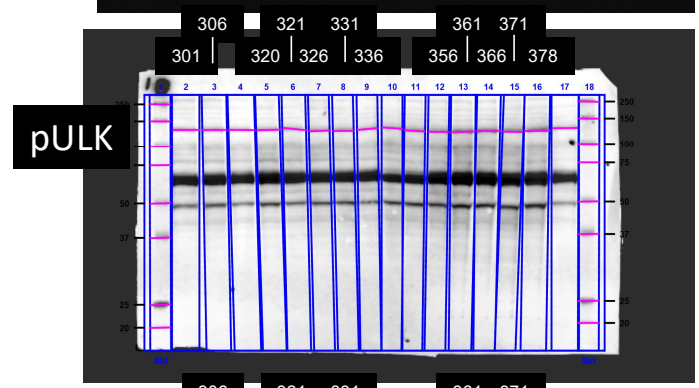

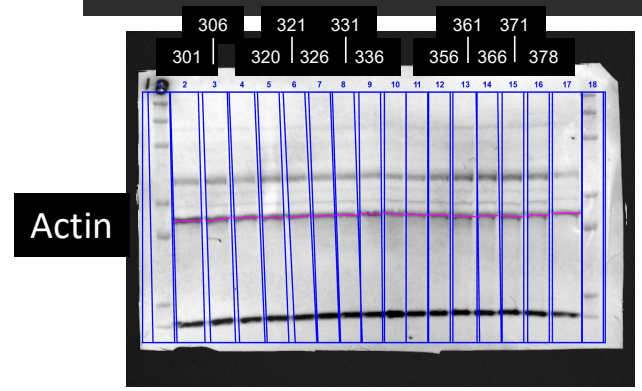

Blot 1C

Blot 1			
Well	Sub#	Sex	Condition
1	Ladder		
2	301	Male	Control
3	306	Male	Obese
4	[redacted]	[redacted]	[redacted]
5	320	Female	Obese+CR
6	321	Female	Control
7	326	Female	Obese
8	331	Male	Obese+CR
9	336	Male	Control+CR
10	[redacted]	[redacted]	[redacted]
11	[redacted]	[redacted]	[redacted]
12	356	Female	Control+CR
13	361	Male	Control+CR
14	366	Male	Obese+CR
15	371	Male	Control
16	378	Female	Obese
17	[redacted]	[redacted]	[redacted]
18	Ladder		

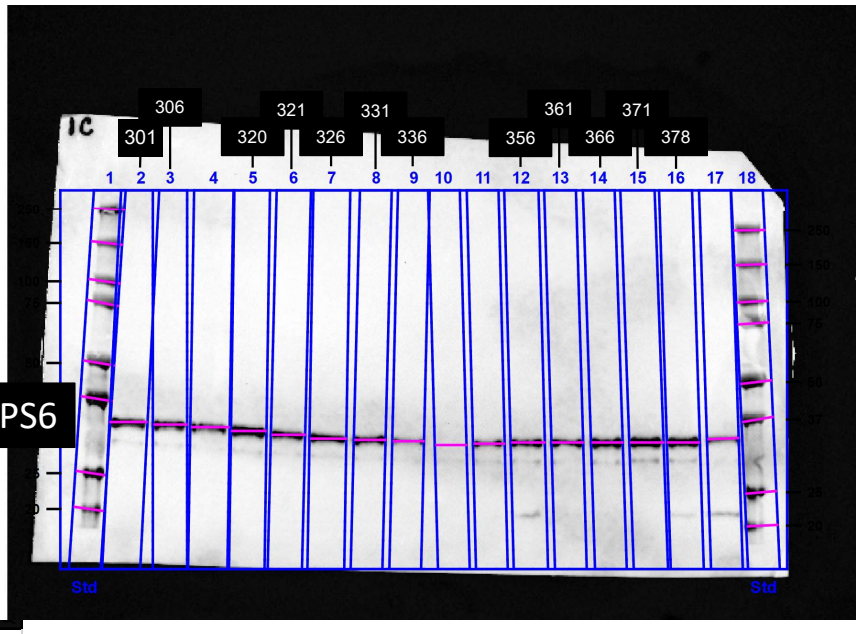

*Blacked out cells are samples from another project that were run alongside these samples, but are not represented in the results of this manuscript.

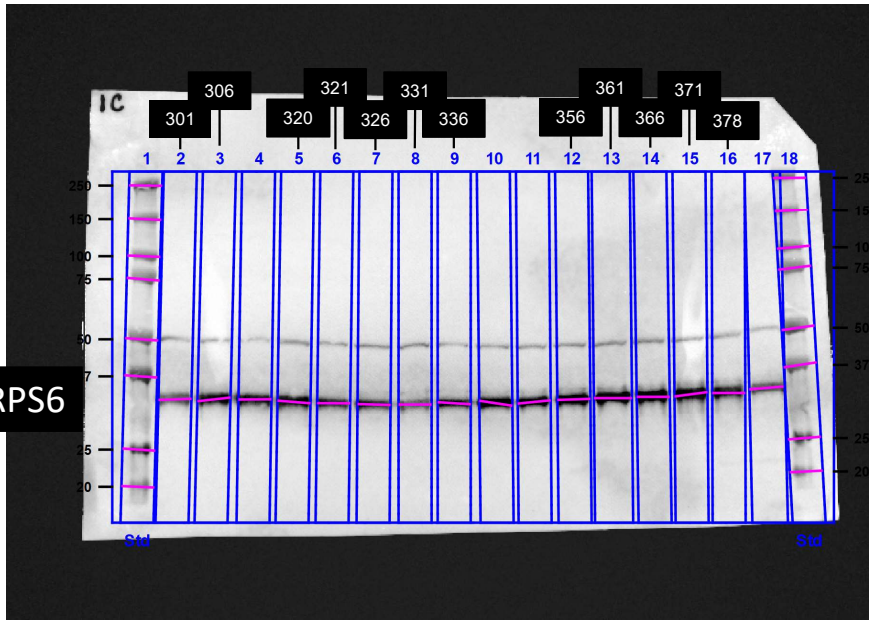

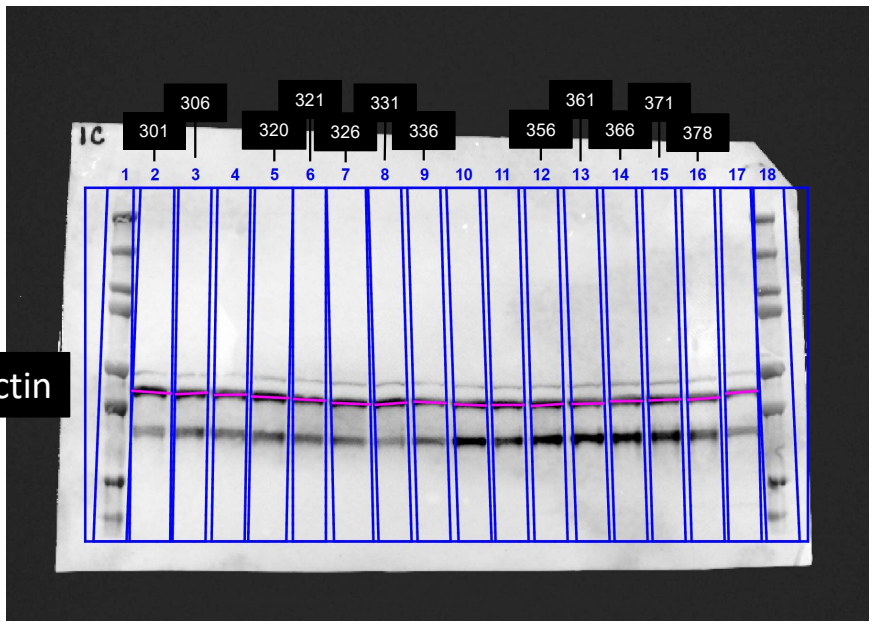

Blot 2A

Blot 2			
Well	Sub#	Sex	Condition
1	Ladder		
2	386	Female	Obese+CR
3	391	Male	Obese
4	396	Male	Chow+CR+CQ
5	402	Male	DIO+CR+CQ
6	406	Female	Control+CR
7	416	Female	Control
8	302	Male	Control
9	308	Male	Obese
10	313	Male	Chow+CR+CQ
11	316	Female	Obese+CR
12	322	Female	Control
13	327	Female	Obese
14	332	Male	Obese+CR
15	337	Male	Control+CR
16	342	Male	DIO+CR+CQ
17	352	Female	DIO+CR+CQ
18	Ladder		

*Blacked out cells are samples from another project that were run alongside these samples, but are not represented in the results of this manuscript.

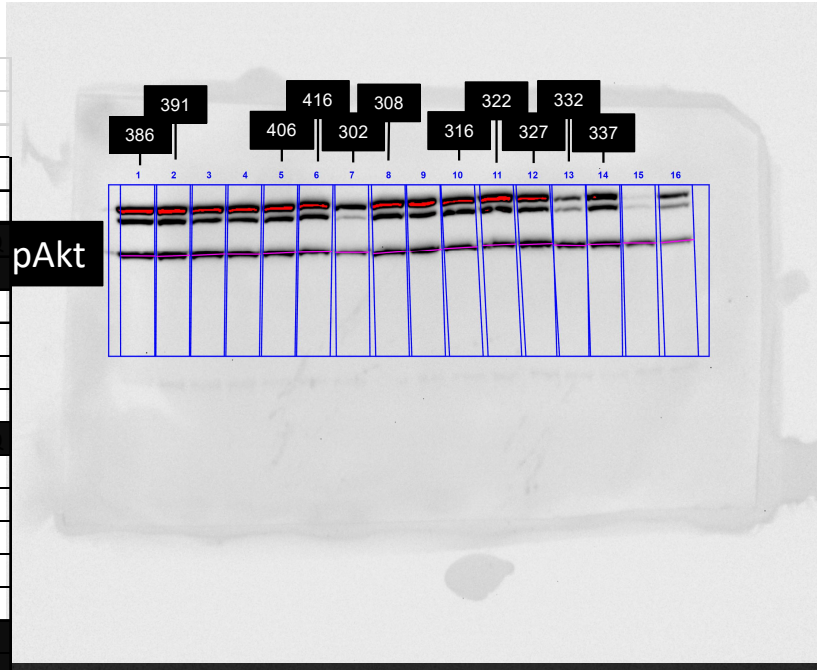

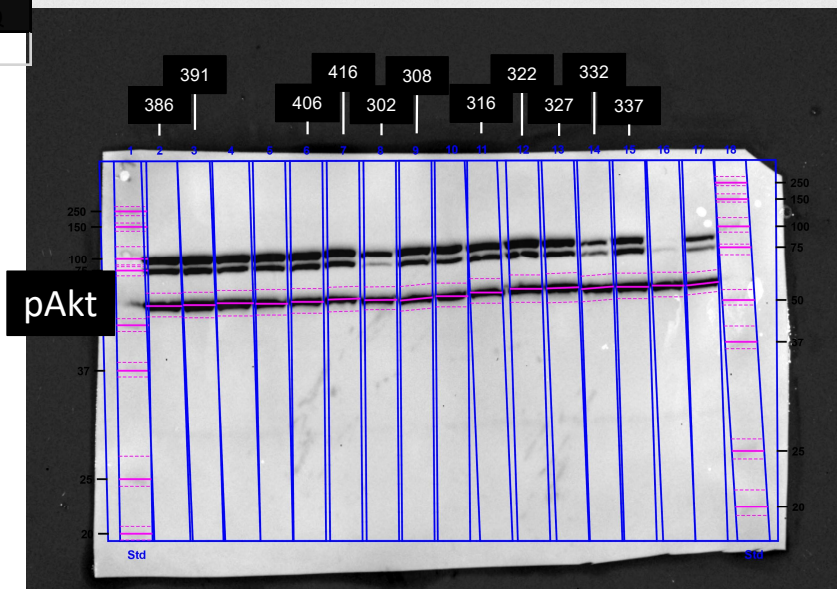

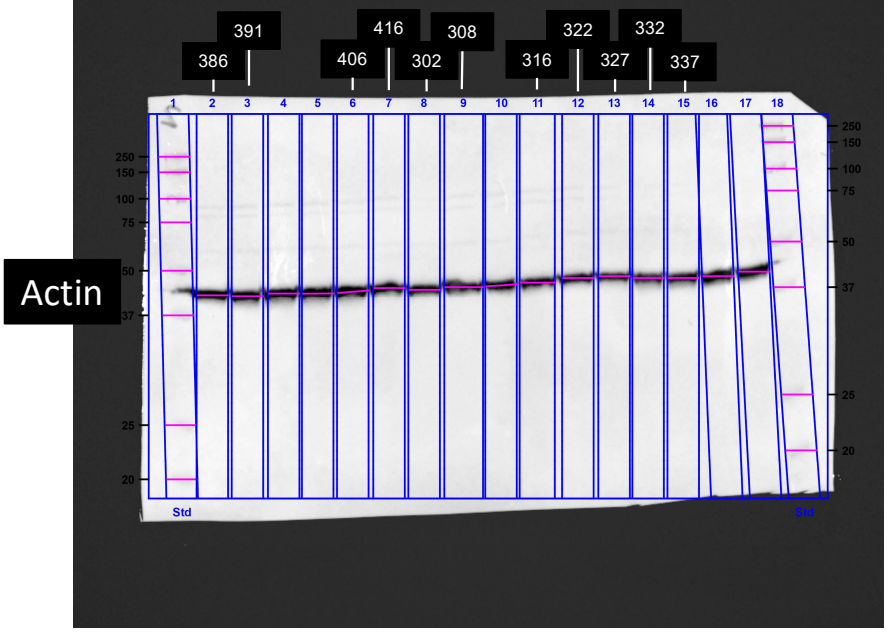

Blot 2B

Blot 2			
Well	Sub#	Sex	Condition
1	Ladder		
2	386	Female	Obese+CR
3	391	Male	Obese
4	396	Male	Chow+CR+CQ
5	402	Male	DIO+CR+CQ
6	406	Female	Control+CR
7	416	Female	Control
8	302	Male	Control
9	308	Male	Obese
10	313	Male	Chow+CR+CQ
11	316	Female	Obese+CR
12	322	Female	Control
13	327	Female	Obese
14	332	Male	Obese+CR
15	337	Male	Control+CR
16	342	Male	DIO+CR+CQ
17	352	Female	DIO+CR+CQ
18	Ladder		

*Blacked out cells are samples from another project that were run alongside these samples, but are not represented in the results of this manuscript.

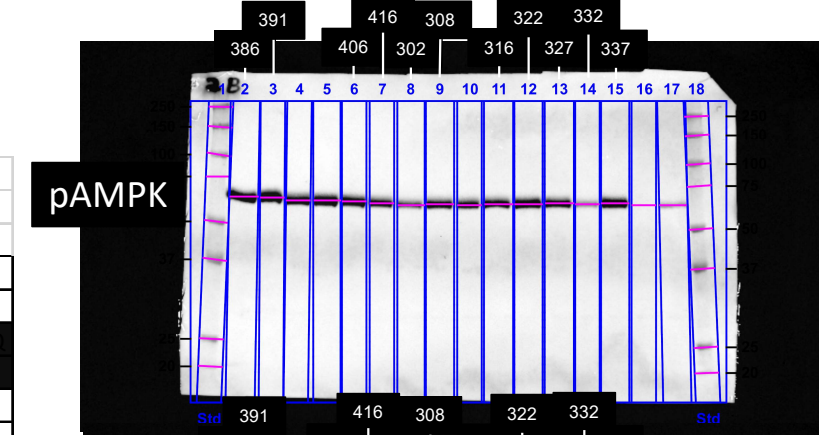

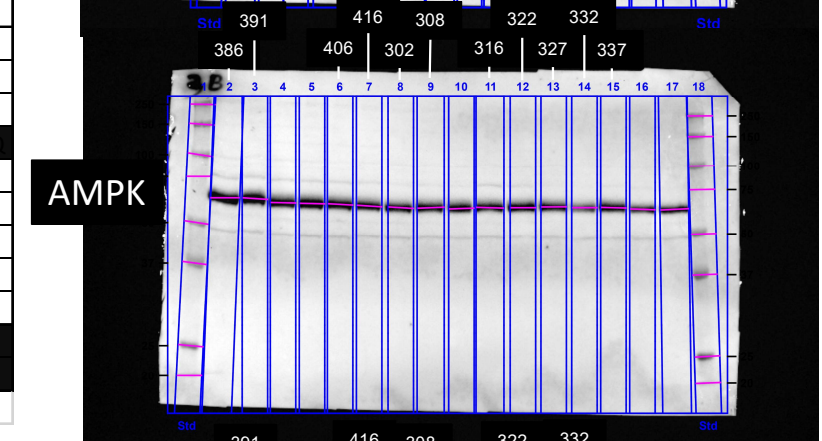

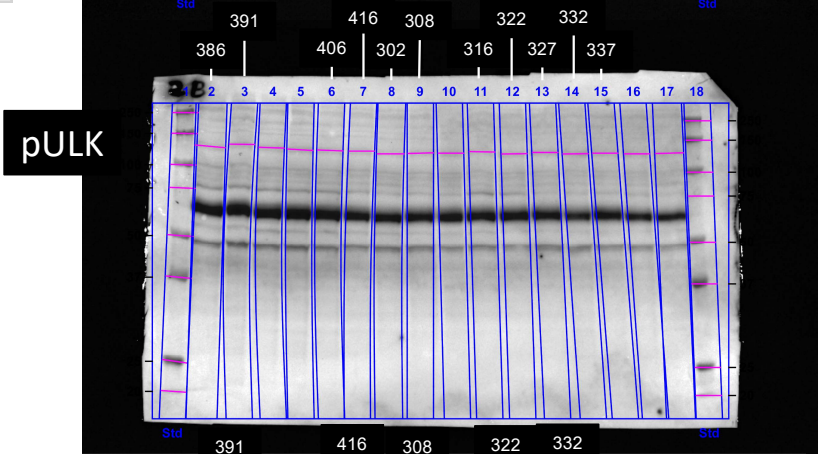

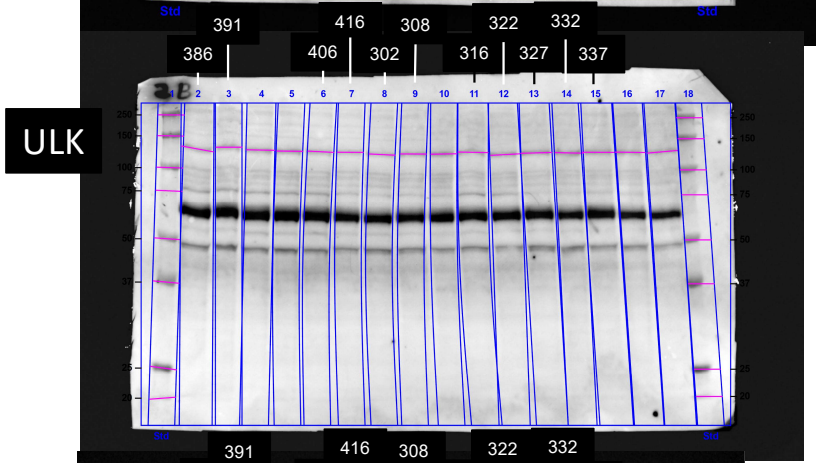

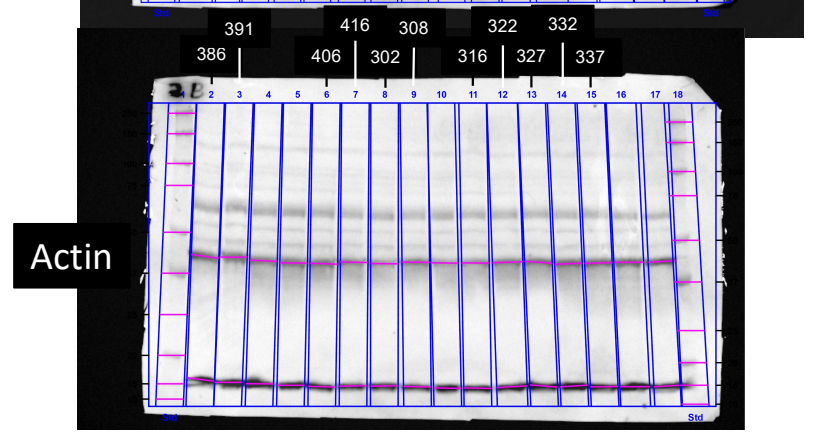

Blot 2C

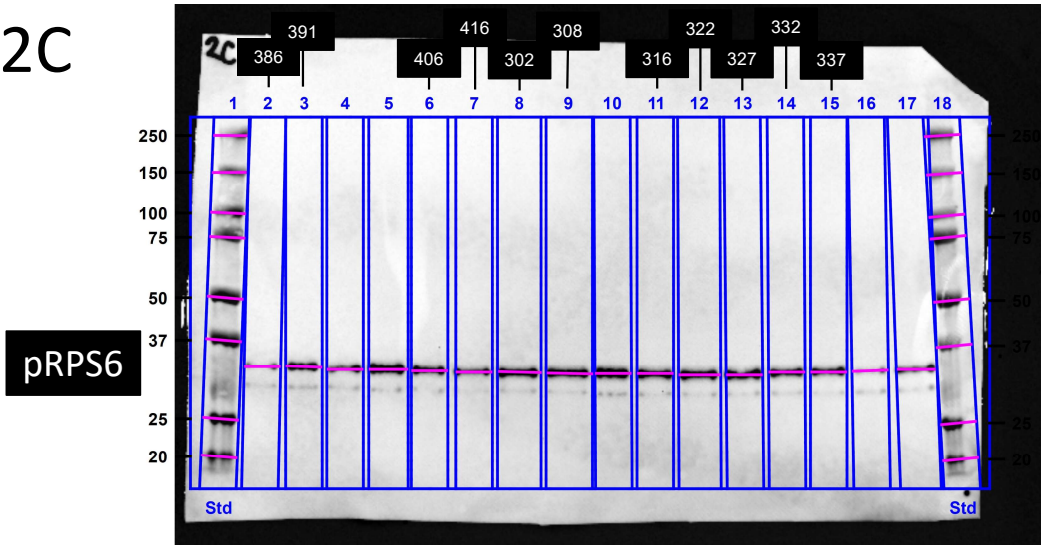

pRPS6

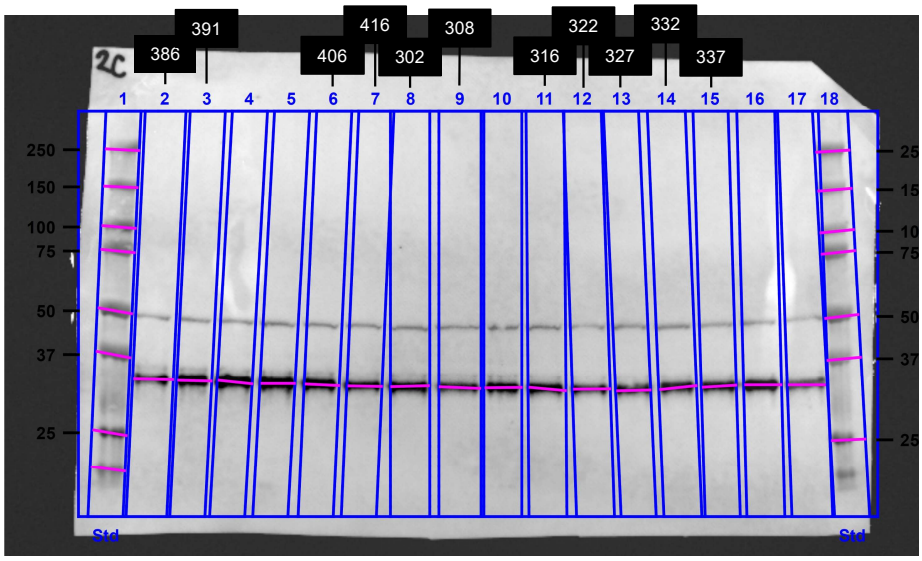

RPS6

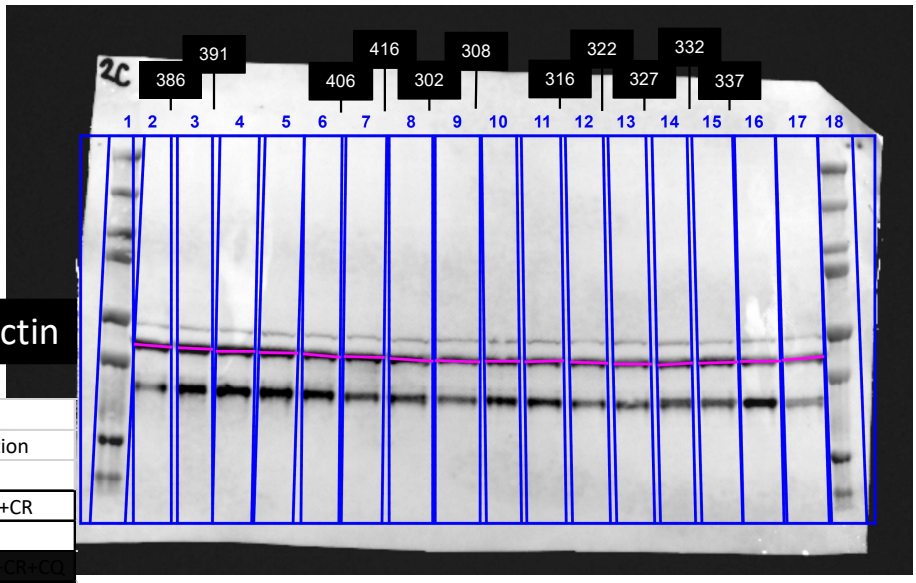

Actin

Blot 2			
Well	Sub#	Sex	Condition
1	Ladder		
2	386	Female	Obese+CR
3	391	Male	Obese
4	396	Male	Chow+CR+CQ
5	402	Male	DIO+CR+CQ
6	406	Female	Control+CR
7	416	Female	Control
8	302	Male	Control
9	308	Male	Obese
10	313	Male	Chow+CR+CQ
11	316	Female	Obese+CR
12	322	Female	Control
13	327	Female	Obese
14	332	Male	Obese+CR
15	337	Male	Control+CR
16	342	Male	DIO+CR+CQ
17	352	Female	DIO+CR+CQ
18	Ladder		

*Blacked out cells are samples from another project that were run alongside these samples, but are not represented in the results of this manuscript.

Blot 3A

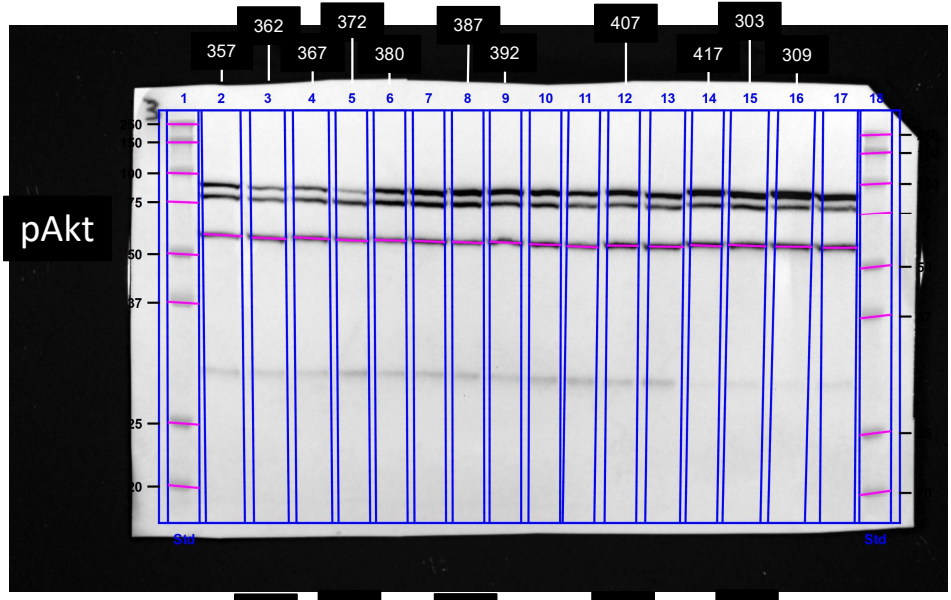

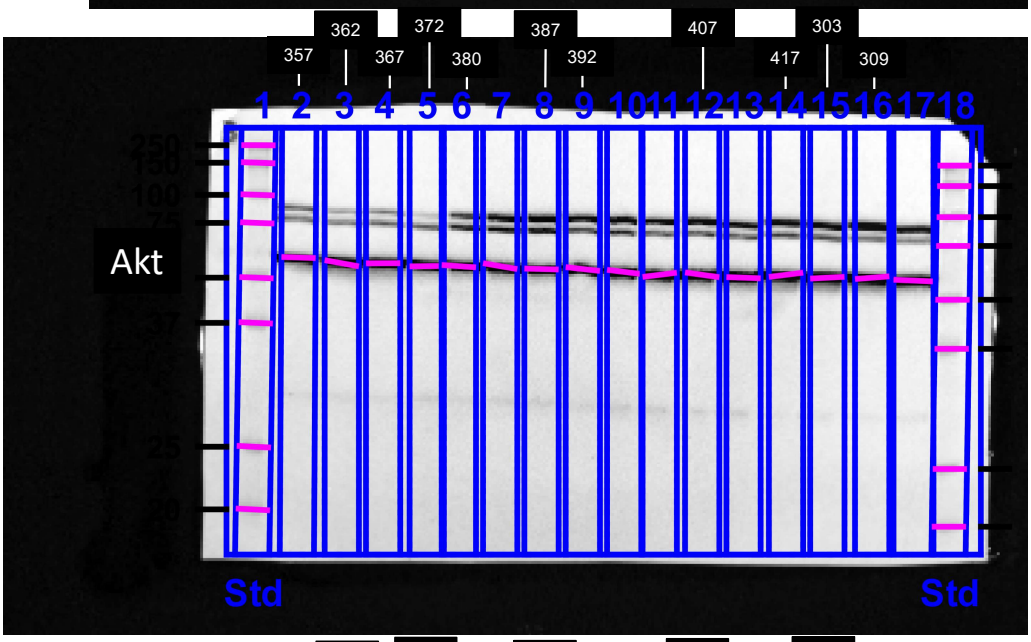

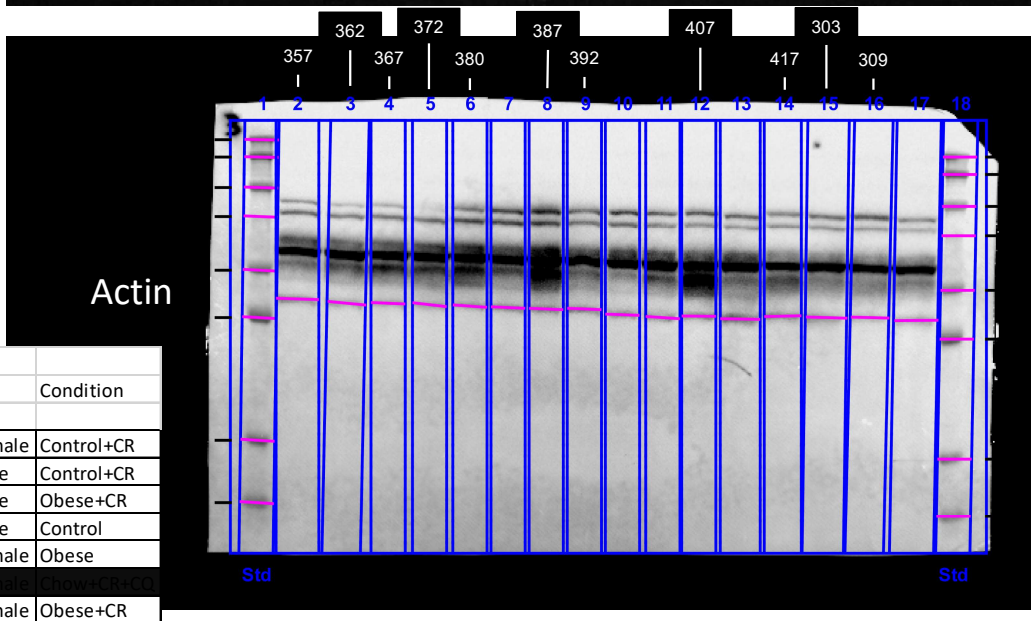

Blot 3			
Well	Sub#	Sex	Condition
1	Ladder		
2	357	Female	Control+CR
3	362	Male	Control+CR
4	367	Male	Obese+CR
5	372	Male	Control
6	380	Female	Obese
7	382	Female	Chow+CR+CO
8	387	Female	Obese+CR
9	392	Male	Obese
10	398	Male	Chow+CR+CO
11	403	Male	DIO+CR+CO
12	407	Female	Chow+CR
13	411	Female	DIO+CR+CO
14	417	Female	Control
15	303	Male	Control
16	309	Male	Obese
17	314	Male	Chow+CR+CO
18	Ladder		

*Blacked out cells are samples from another project that were run alongside these samples, but are not represented in the results of this manuscript.

Blot 3B

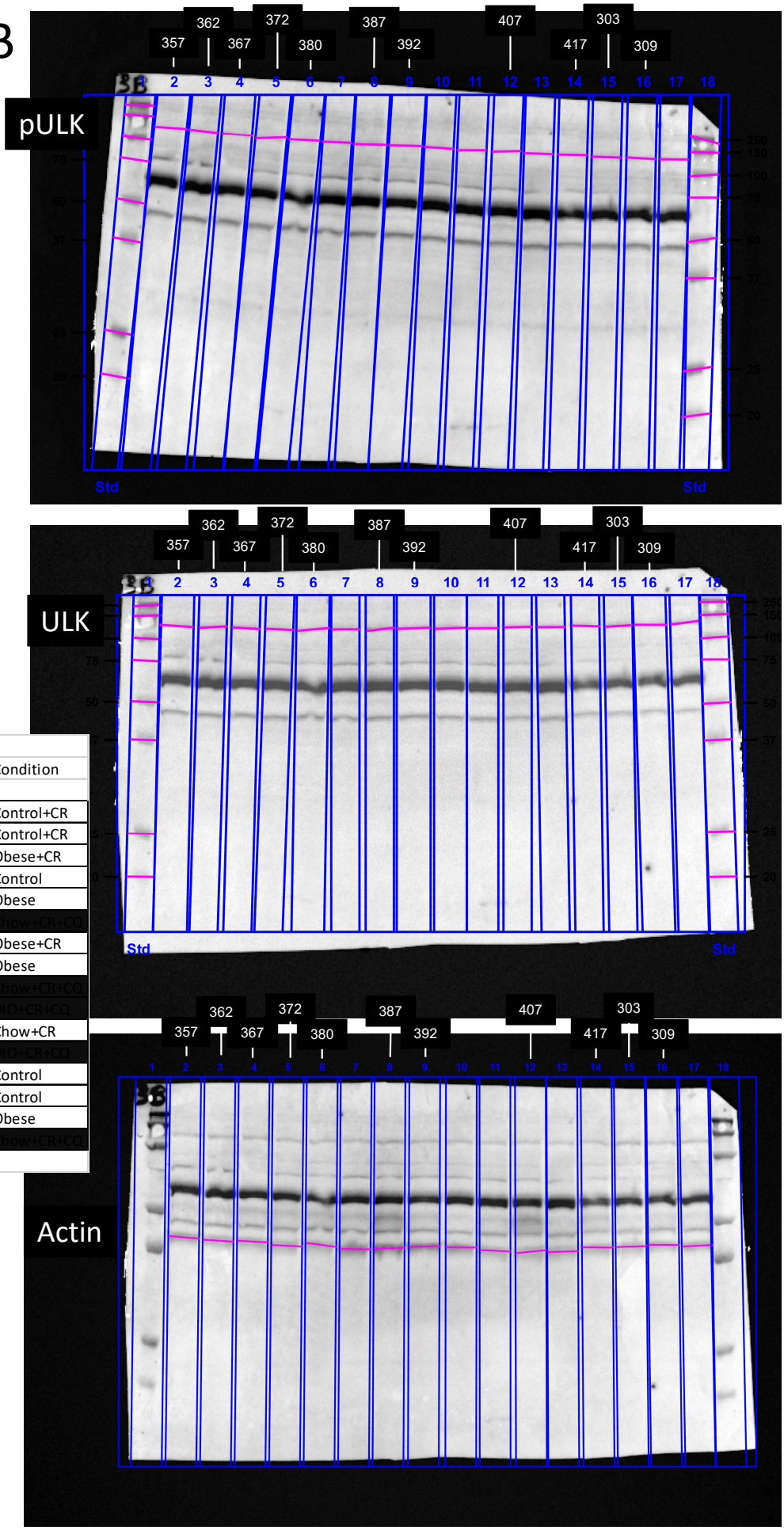

Blot 3			
Well	Sub#	Sex	Condition
1	Ladder		
2	357	Female	Control+CR
3	362	Male	Control+CR
4	367	Male	Obese+CR
5	372	Male	Control
6	380	Female	Obese
7	382	Female	Chow+CR+CQ
8	387	Female	Obese+CR
9	392	Male	Obese
10	398	Male	Chow+CR+CQ
11	403	Male	DIO+CR+CQ
12	407	Female	Chow+CR
13	411	Female	DIO+CR+CQ
14	417	Female	Control
15	303	Male	Control
16	309	Male	Obese
17	314	Male	Chow+CR+CQ
18	Ladder		

*Blacked out cells are samples from another project that were run alongside these samples, but are not represented in the results of this manuscript.

Blot 3C

Blot 3			
Well	Sub#	Sex	Condition
1	Ladder		
2	357	Female	Control+CR
3	362	Male	Control+CR
4	367	Male	Obese+CR
5	372	Male	Control
6	380	Female	Obese
7	382	Female	Chow+CR+CQ
8	387	Female	Obese+CR
9	392	Male	Obese
10	398	Male	Chow+CR+CQ
11	403	Male	DIO+CR+CQ
12	407	Female	Chow+CR
13	411	Female	DIO+CR+CQ
14	417	Female	Control
15	303	Male	Control
16	309	Male	Obese
17	314	Male	Chow+CR+CQ
18	Ladder		

*Blacked out cells are samples from another project that were run alongside these samples, but are not represented in the results of this manuscript.

pAMPK

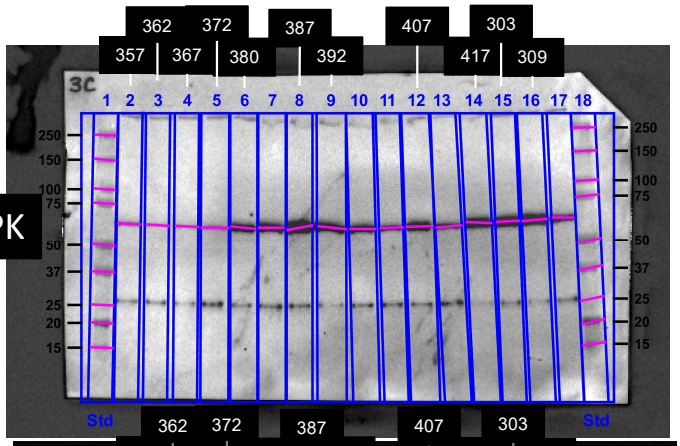

AMPK

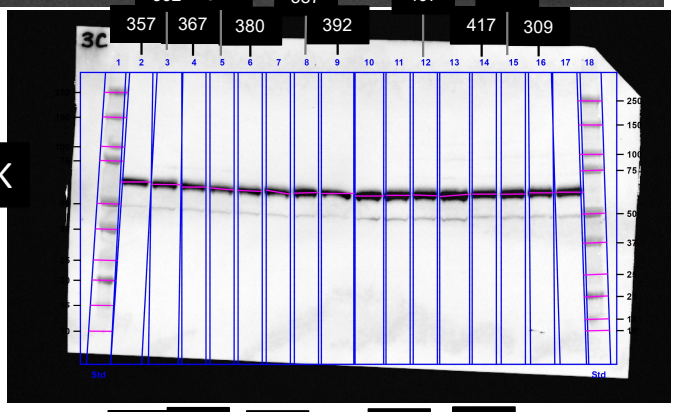

pRPS6

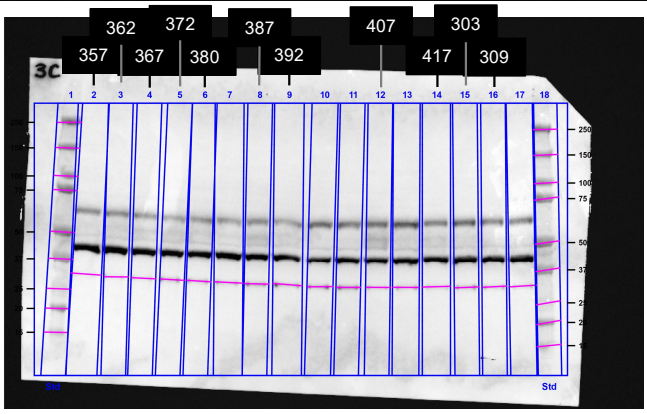

RPS6

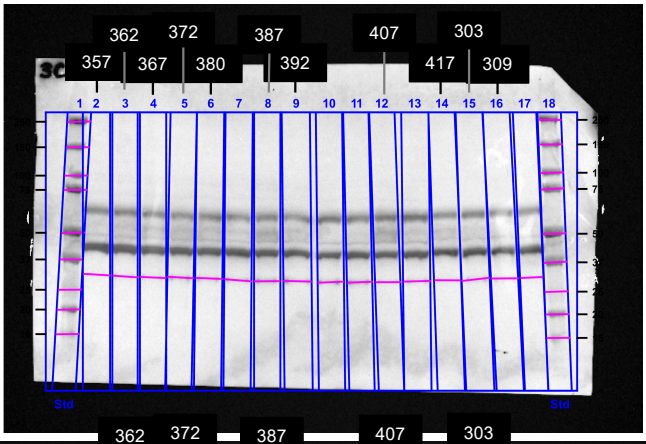

Actin

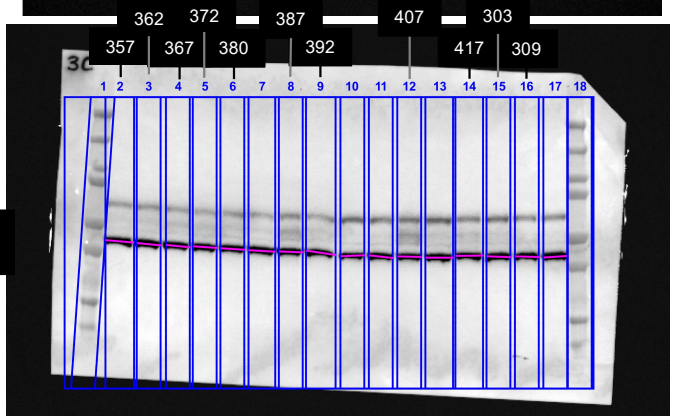

Blot 4A

pAkt

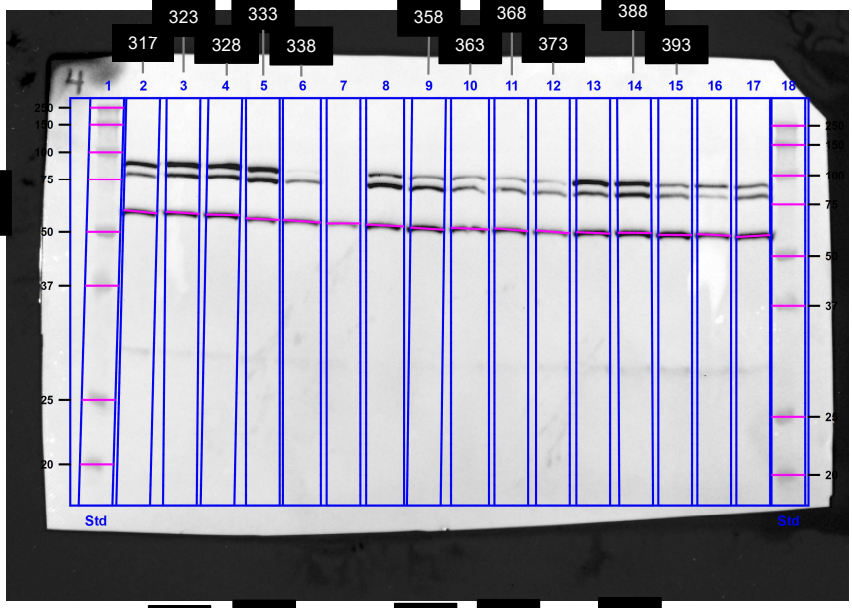

Akt

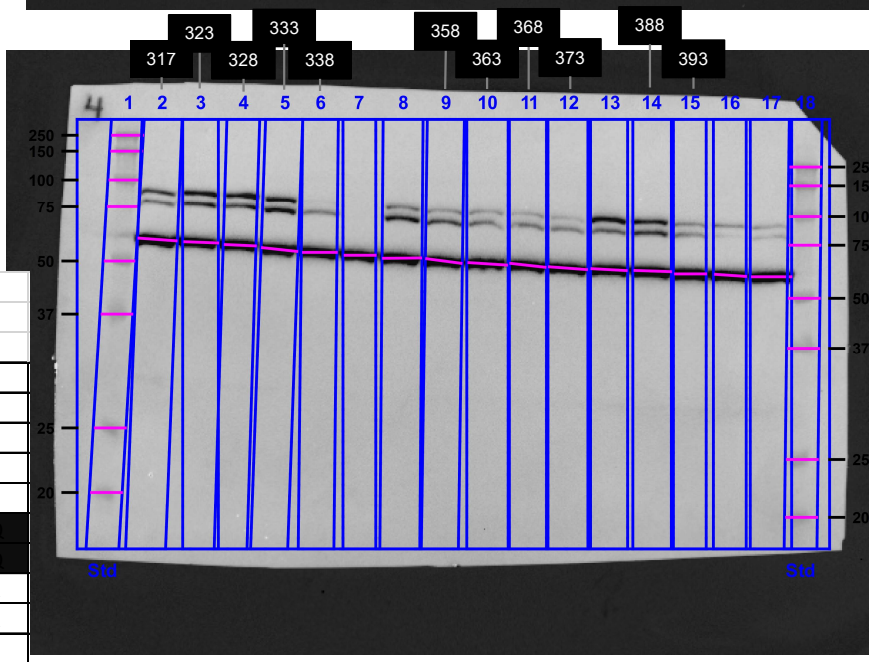

Blotl 4			
Well	Sub#	Sex	Condition
1	Ladder		
2	317	Female	Obese+CR
3	323	Female	Control
4	328	Female	Obese
5	333	Male	Obese+CR
6	338	Male	Chow+CR
7	343	Male	DIO+CR+CQ
8	353	Female	DIO+CR+CQ
9	358	Female	Control+CR
10	363	Male	Control+CR
11	368	Male	Obese+CR
12	373	Male	Control
13	383	Female	Chow+CR+CQ
14	388	Female	Obese+CR
15	393	Male	Obese
16	400	Male	Chow+CR+CQ
17	404	Male	DIO+CR+CQ
18	Ladder		

Actin

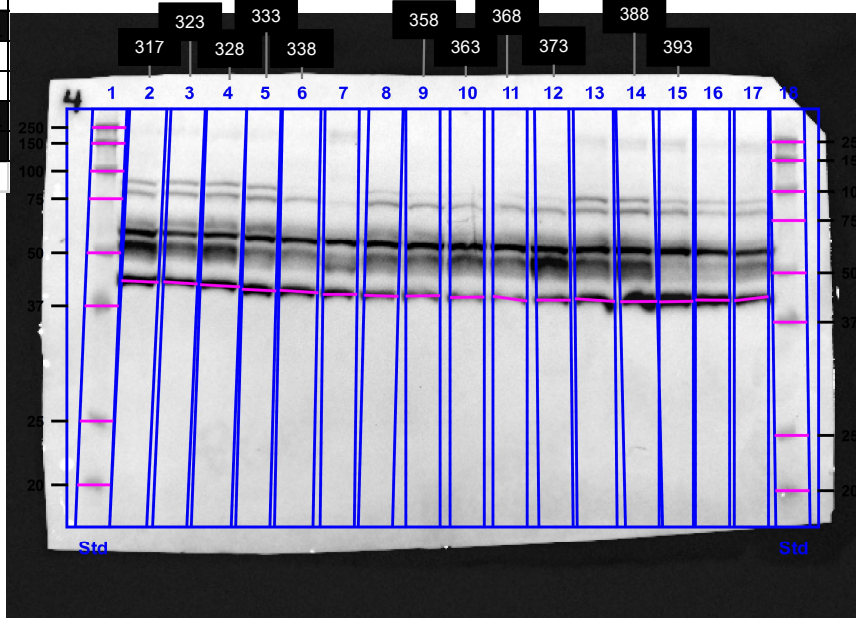

*Blacked out cells are samples from another project that were run alongside these samples, but are not represented in the results of this manuscript.

Blot 4B

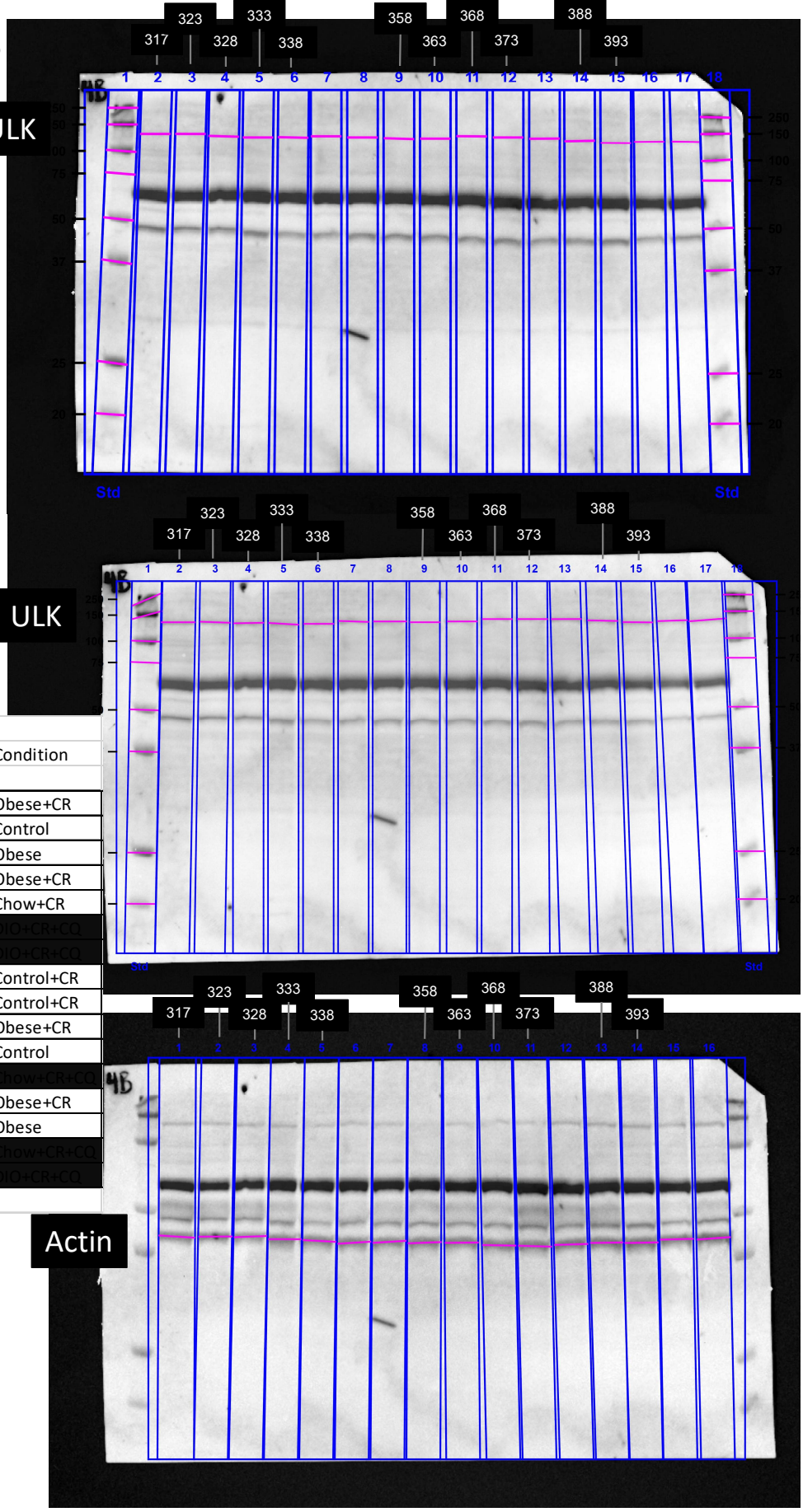

Blotl 4			
Well	Sub#	Sex	Condition
1	Ladder		
2	317	Female	Obese+CR
3	323	Female	Control
4	328	Female	Obese
5	333	Male	Obese+CR
6	338	Male	Chow+CR
7	343	Male	DIO+CR+CQ
8	353	Female	DIO+CR+CQ
9	358	Female	Control+CR
10	363	Male	Control+CR
11	368	Male	Obese+CR
12	373	Male	Control
13	383	Female	Chow+CR+CQ
14	388	Female	Obese+CR
15	393	Male	Obese
16	400	Male	Chow+CR+CQ
17	404	Male	DIO+CR+CQ
18	Ladder		

*Blacked out cells are samples from another project that were run alongside these samples, but are not represented in the results of this manuscript.

Blot 4C

Blotl 4			
Well	Sub#	Sex	Condition
1	Ladder		
2	317	Female	Obese+CR
3	323	Female	Control
4	328	Female	Obese
5	333	Male	Obese+CR
6	338	Male	Chow+CR
7	343	Male	DIO+CR+CQ
8	353	Female	DIO+CR+CQ
9	358	Female	Control+CR
10	363	Male	Control+CR
11	368	Male	Obese+CR
12	373	Male	Control
13	383	Female	Chow+CR+CQ
14	388	Female	Obese+CR
15	393	Male	Obese
16	400	Male	Chow+CR+CQ
17	404	Male	DIO+CR+CQ
18	Ladder		

*Blacked out cells are samples from another project that were run alongside these samples, but are not represented in the results of this manuscript.

pAMPK

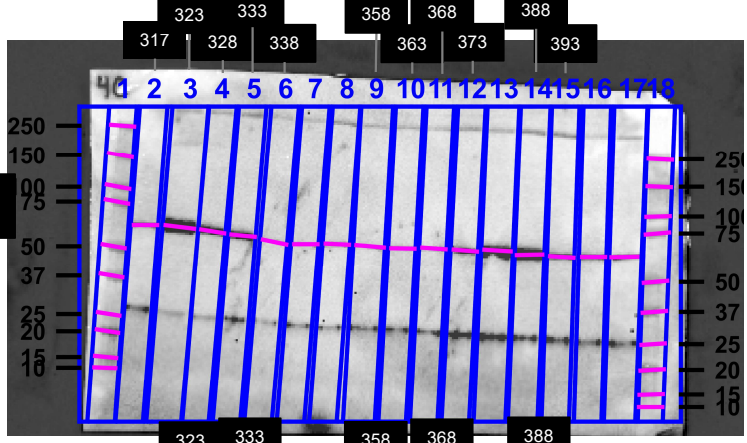

AMPK

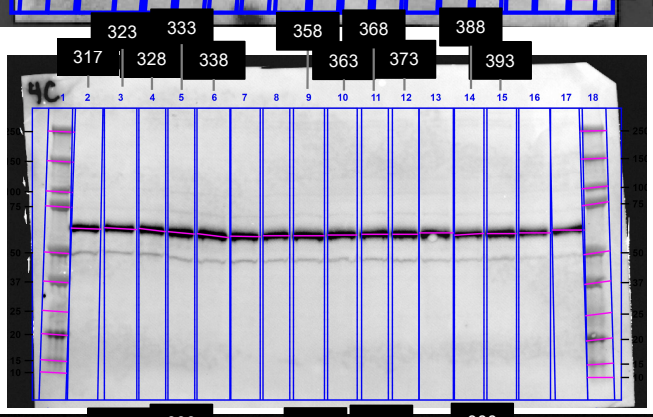

pRPS6

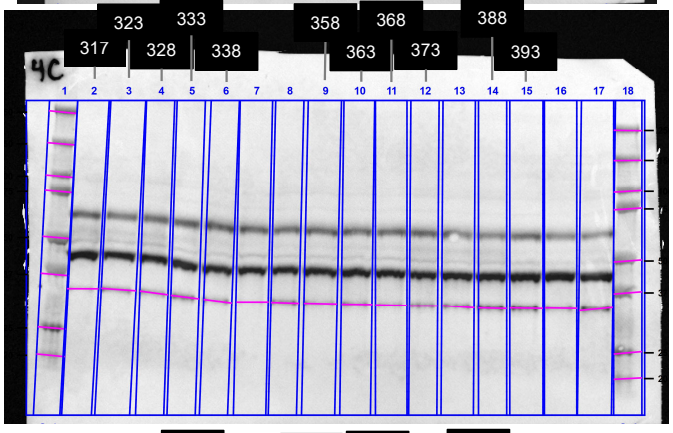

RPS6

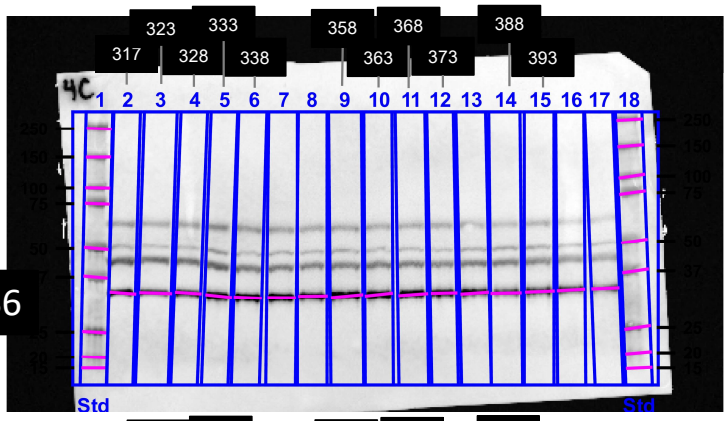

Actin

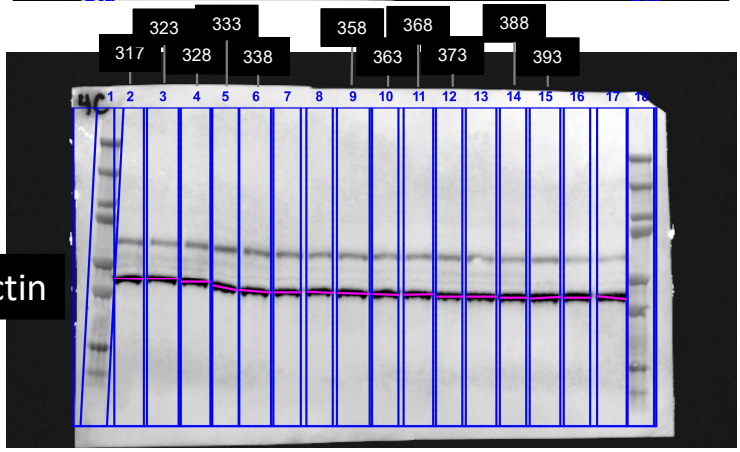

Blot 5A

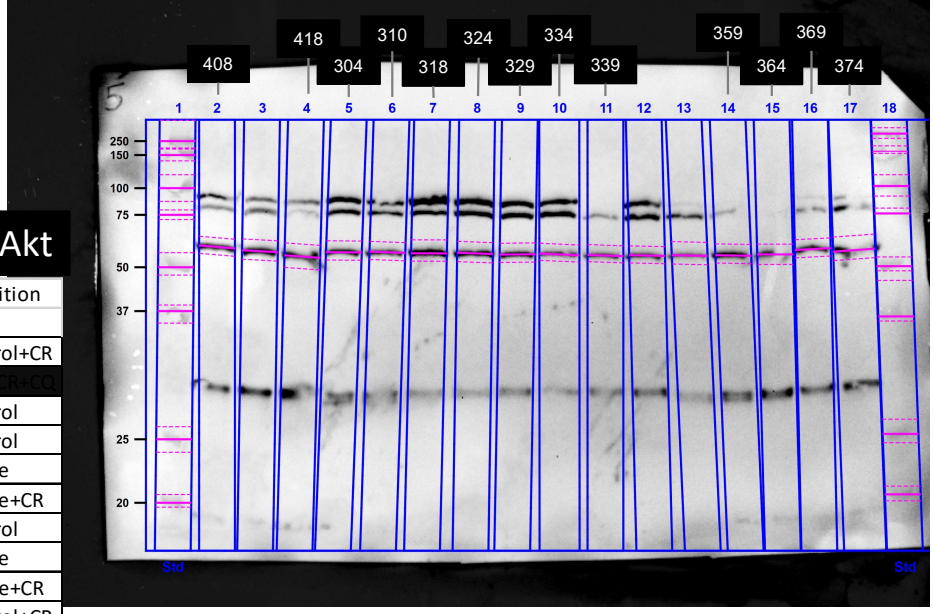

Well	Sub#	Sex	Condition
1	Ladder		
2	408	Female	Control+CR
3	413	Female	DIO+CR+CO
4	418	Female	Control
5	304	Male	Control
6	310	Male	Obese
7	318	Female	Obese+CR
8	324	Female	Control
9	329	Female	Obese
10	334	Male	Obese+CR
11	339	Male	Control+CR
12	344	Male	DIO+CR+CO
13	354	Female	DIO+CR+CO
14	359	Female	Control+CR
15	364	Male	Control+CR
16	369	Male	Obese+CR
17	374	Male	Control
18	Ladder		

*Blacked out cells are samples from another project that were run alongside these samples, but are not represented in the results of this manuscript.

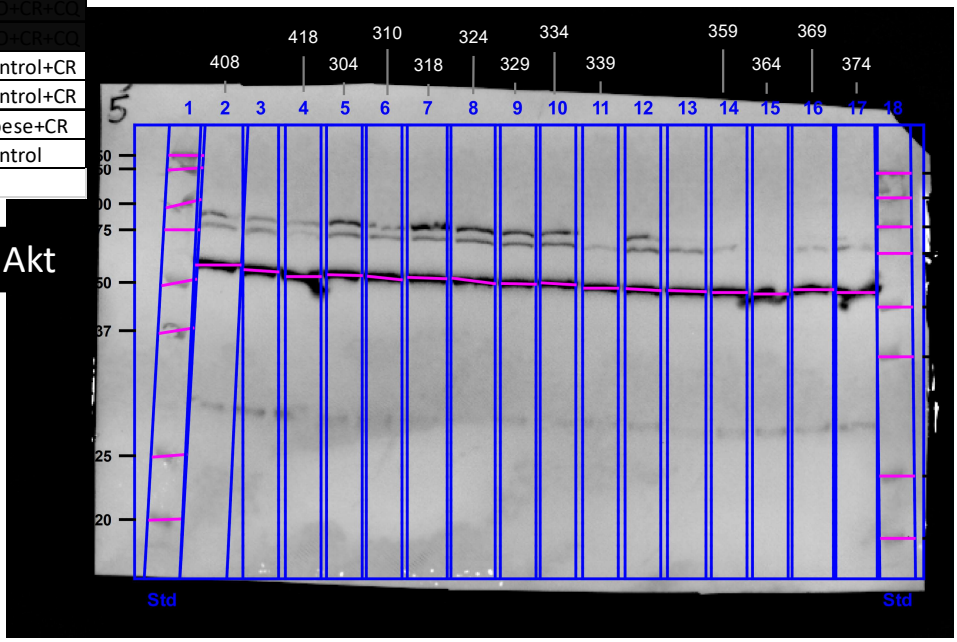

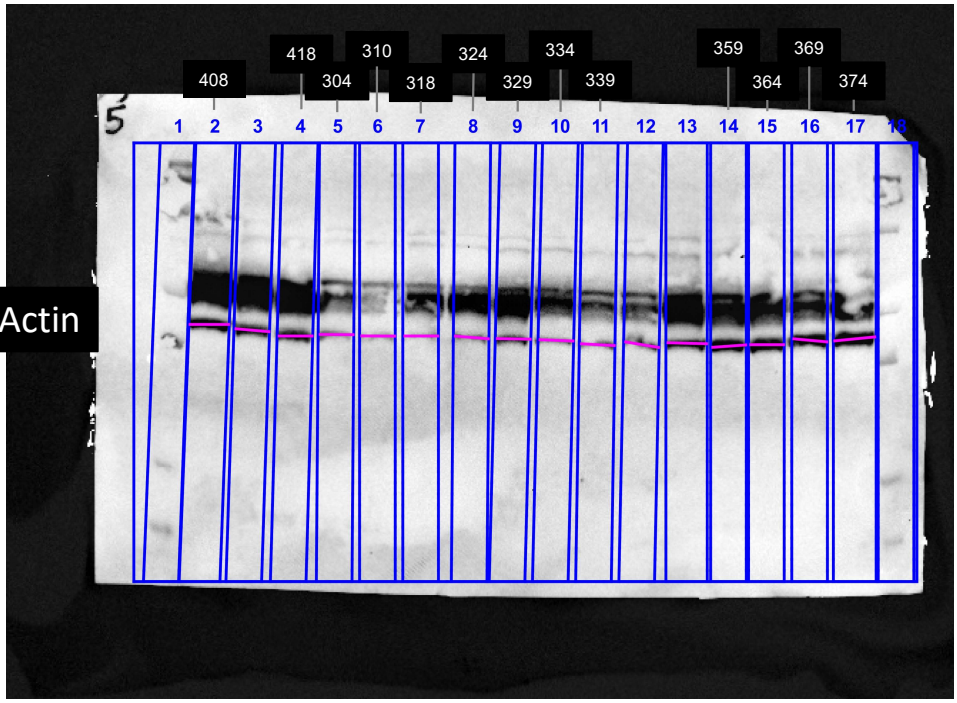

Blot 5B

Well	Sub#	Sex	Condition
1	Ladder		
2	408	Female	Control+CR
3	413	Female	DIO+CR+CQ
4	418	Female	Control
5	304	Male	Control
6	310	Male	Obese
7	318	Female	Obese+CR
8	324	Female	Control
9	329	Female	Obese
10	334	Male	Obese+CR
11	339	Male	Control+CR
12	344	Male	DIO+CR+CQ
13	354	Female	DIO+CR+CQ
14	359	Female	Control+CR
15	364	Male	Control+CR
16	369	Male	Obese+CR
17	374	Male	Control
18	Ladder		

*Blacked out cells are samples from another project that were run alongside these samples, but are not represented in the results of this manuscript.

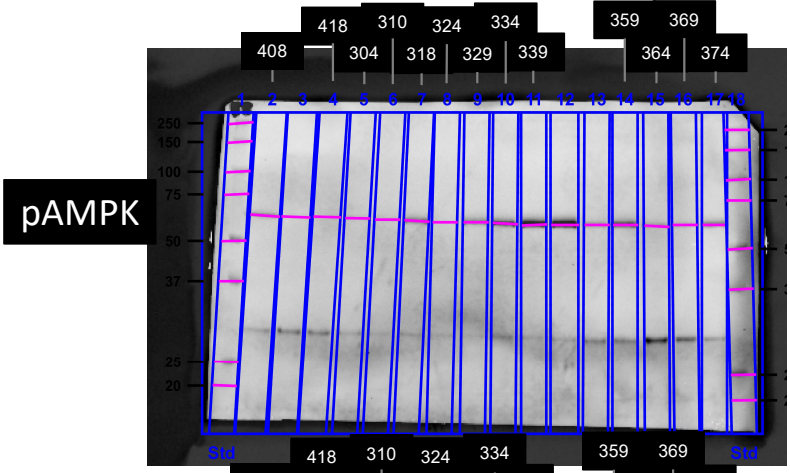

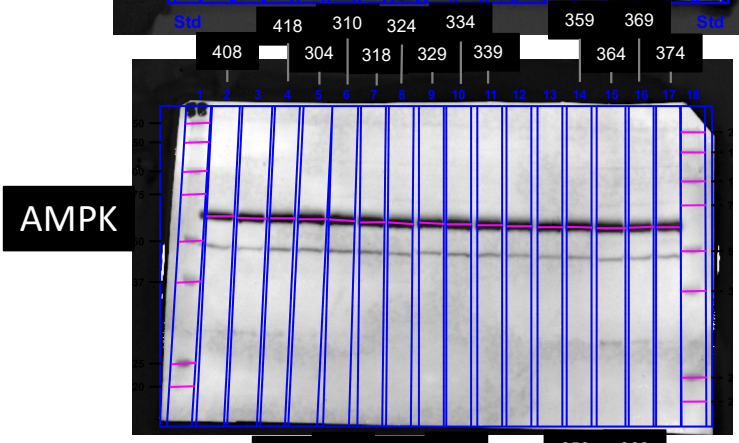

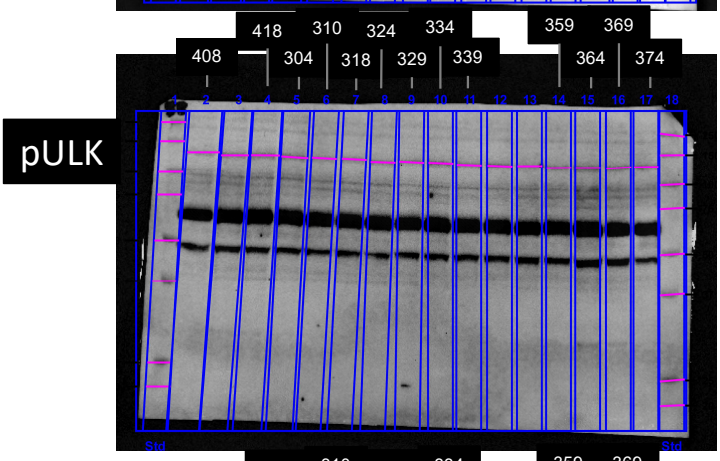

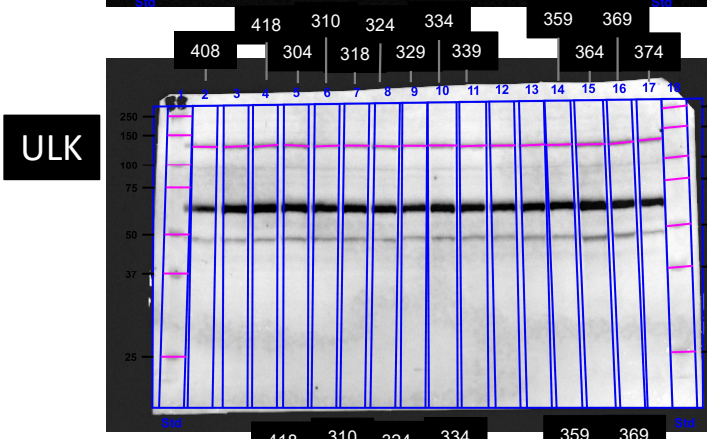

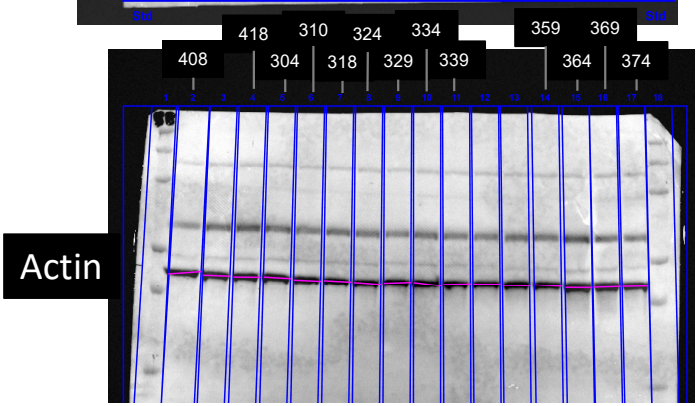

Blot 5C

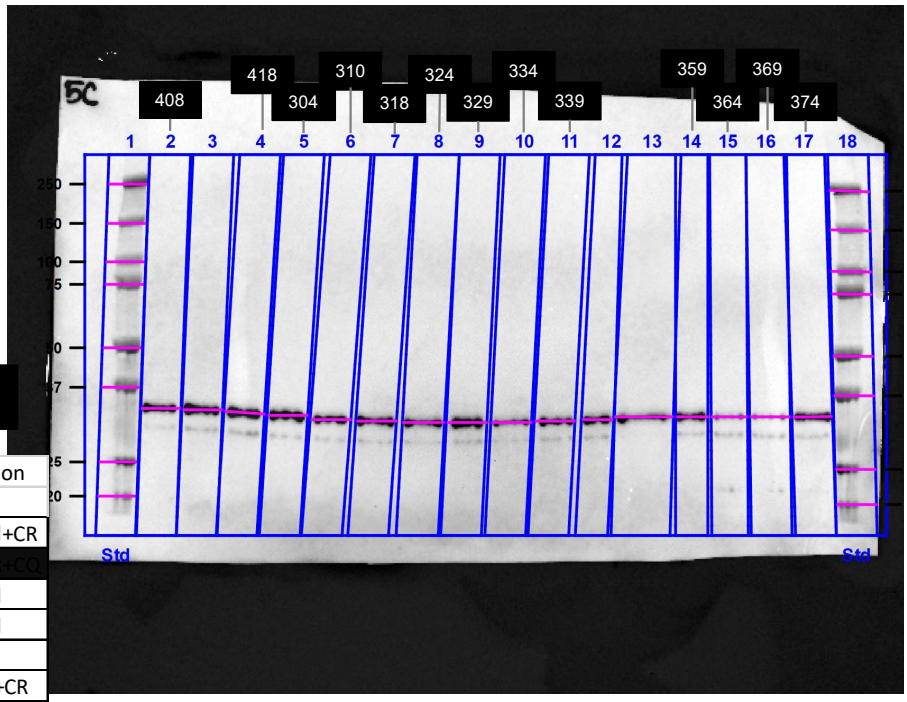

pRPS6

Well	Sub#	Sex	Condition
1	Ladder		
2	408	Female	Control+CR
3	413	Female	DIO+CR+CQ
4	418	Female	Control
5	304	Male	Control
6	310	Male	Obese
7	318	Female	Obese+CR
8	324	Female	Control
9	329	Female	Obese
10	334	Male	Obese+CR
11	339	Male	Control+CR
12	344	Male	DIO+CR+CQ
13	354	Female	DIO+CR+CQ
14	359	Female	Control+CR
15	364	Male	Control+CR
16	369	Male	Obese+CR
17	374	Male	Control
18	Ladder		

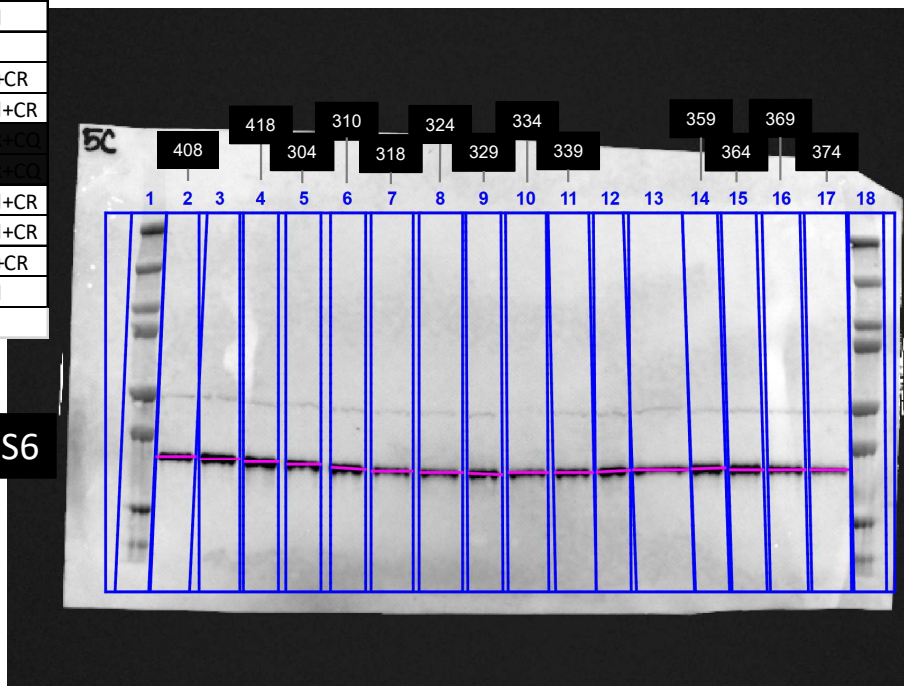

RPS6

*Blacked out cells are samples from another project that were run alongside these samples, but are not represented in the results of this manuscript.

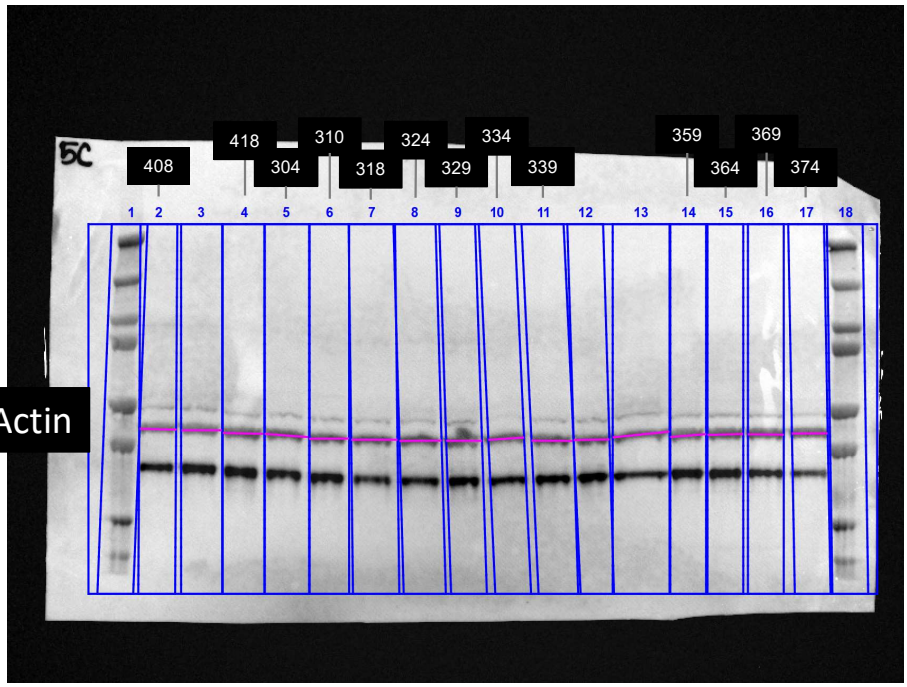

Actin

Blot 6A

Blot 6 Well	Sub#	Sex	Condition
1	Ladder		
2	384	Female	Chow+CR+CQ
3	389	Female	Obese+CR
4	409	Female	Control+CR
5	414	Female	DIO+CR+CQ
6	419	Female	Control
7	305	Male	Control
8	325	Female	Control
9	330	Female	Obese
10	335	Male	Obese+CR
11	340	Male	Control+CR
12	345	Male	DIO+CR+CQ
13	355	Female	DIO+CR+CQ
14	360	Female	Control+CR
15	365	Male	Control+CR
16	370	Male	Obese+CR
17	375	Male	Control
18	Ladder		

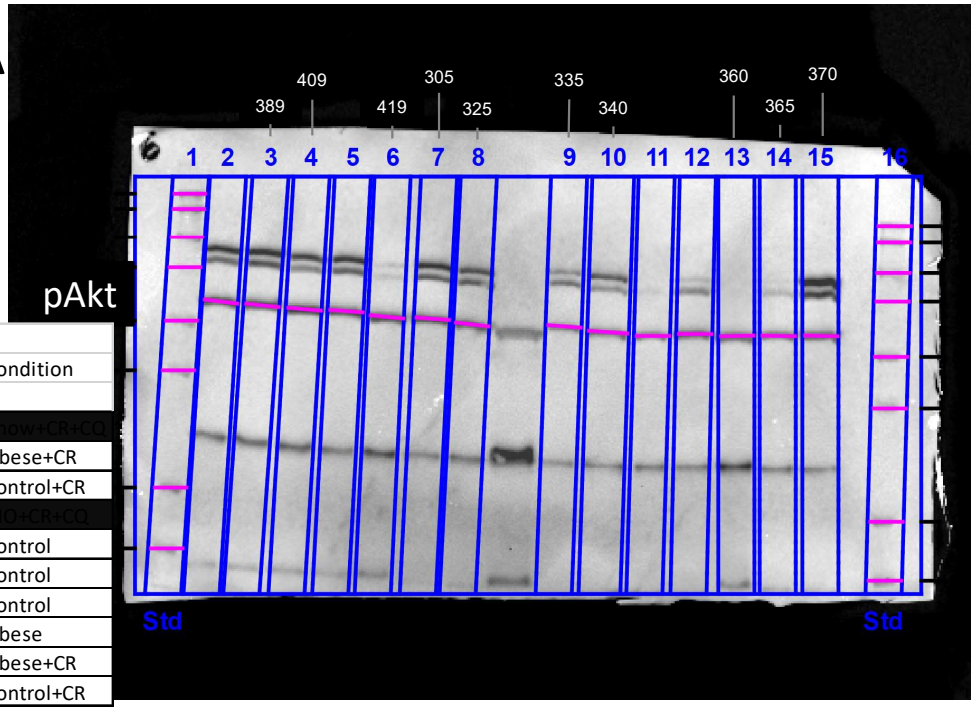

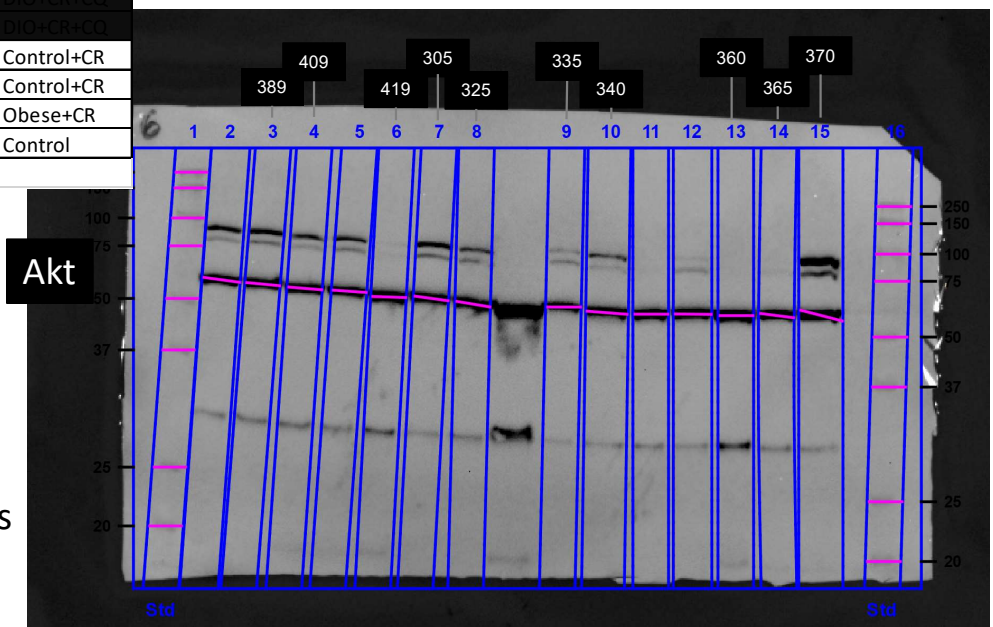

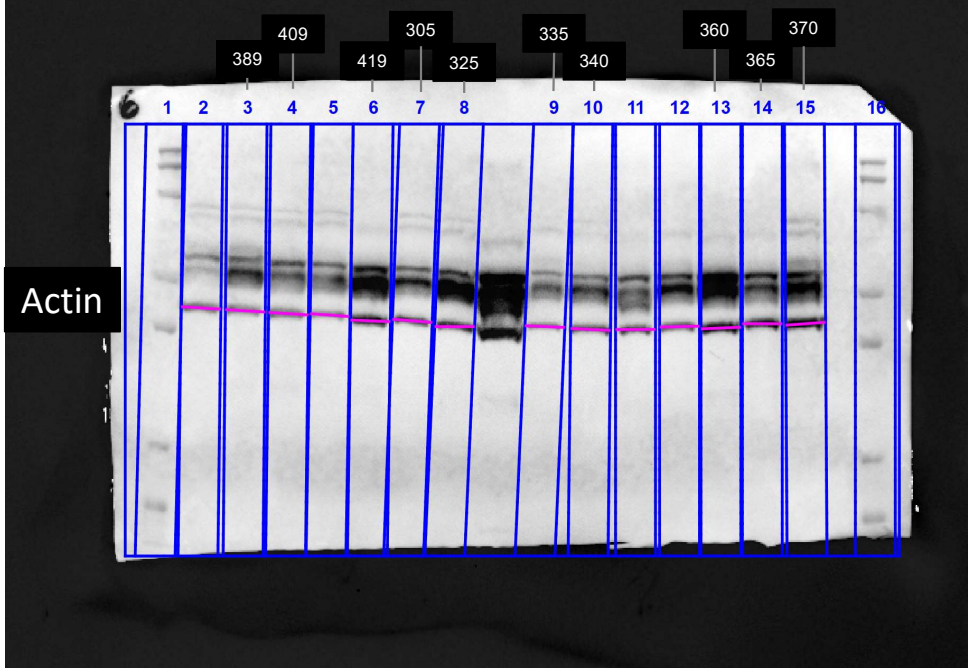

*Blacked out cells are samples from another project that were run alongside these samples, but are not represented in the results of this manuscript.

Blot 6B

Blot 6			
Well	Sub#	Sex	Condition
1	Ladder		
2	384	Female	Chow+CR+CQ
3	389	Female	Obese+CR
4	409	Female	Control+CR
5	414	Female	DIO+CR+CQ
6	419	Female	Control
7	305	Male	Control
8	325	Female	Control
9	330	Female	Obese
10	335	Male	Obese+CR
11	340	Male	Control+CR
12	345	Male	DIO+CR+CQ
13	355	Female	DIO+CR+CQ
14	360	Female	Control+CR
15	365	Male	Control+CR
16	370	Male	Obese+CR
17	375	Male	Control
18	Ladder		

*Blacked out cells are samples from another project that were run alongside these samples, but are not represented in the results of this manuscript.

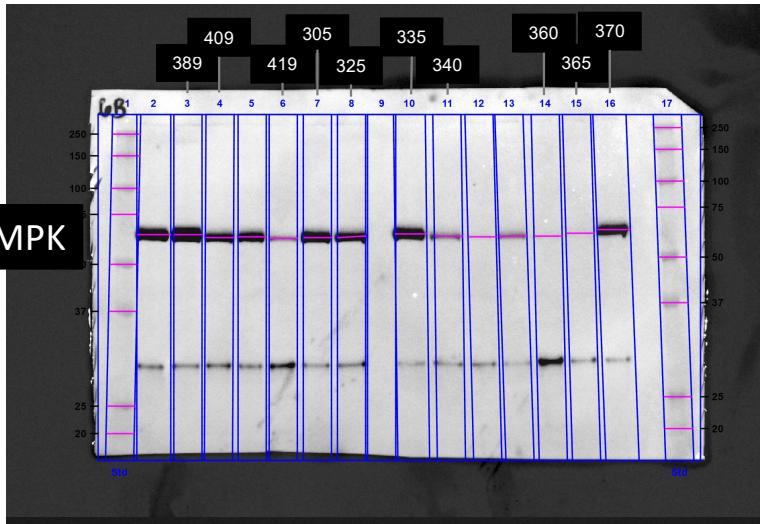

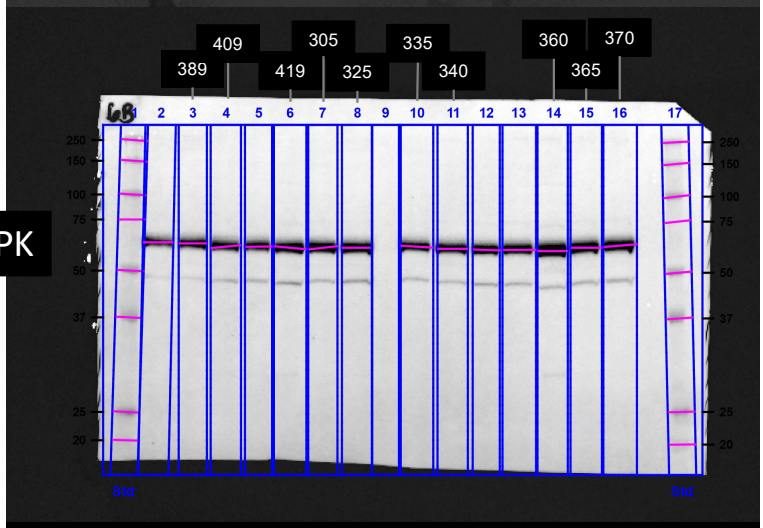

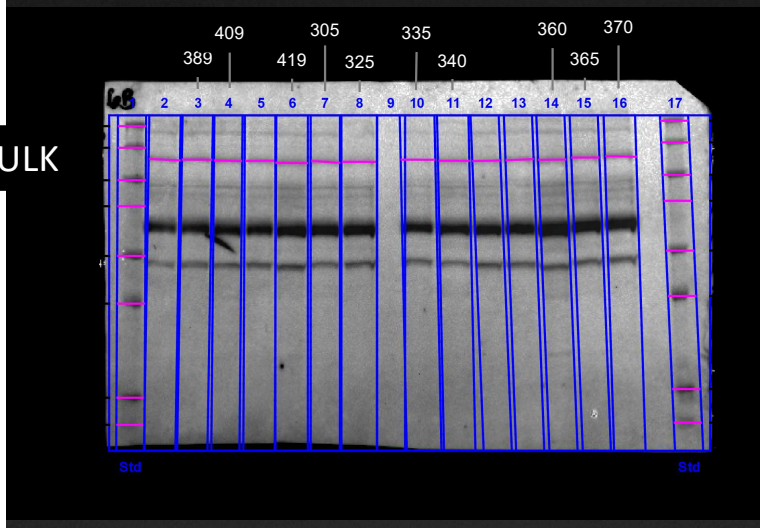

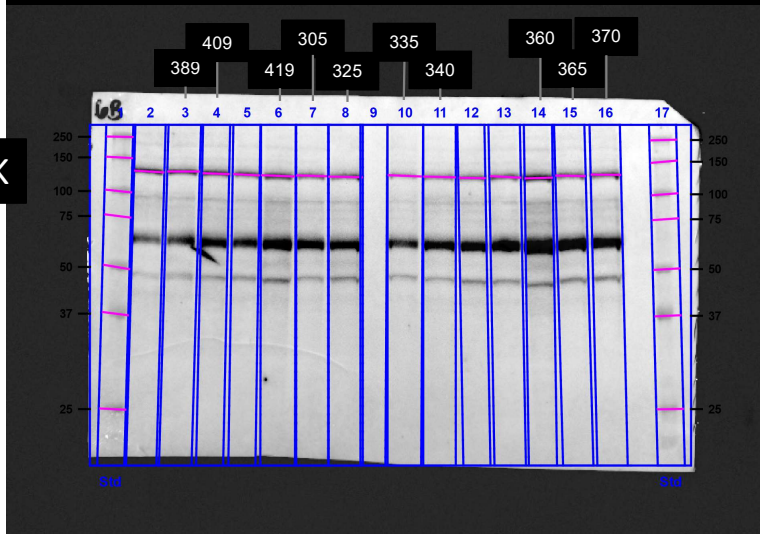

Blot 6B

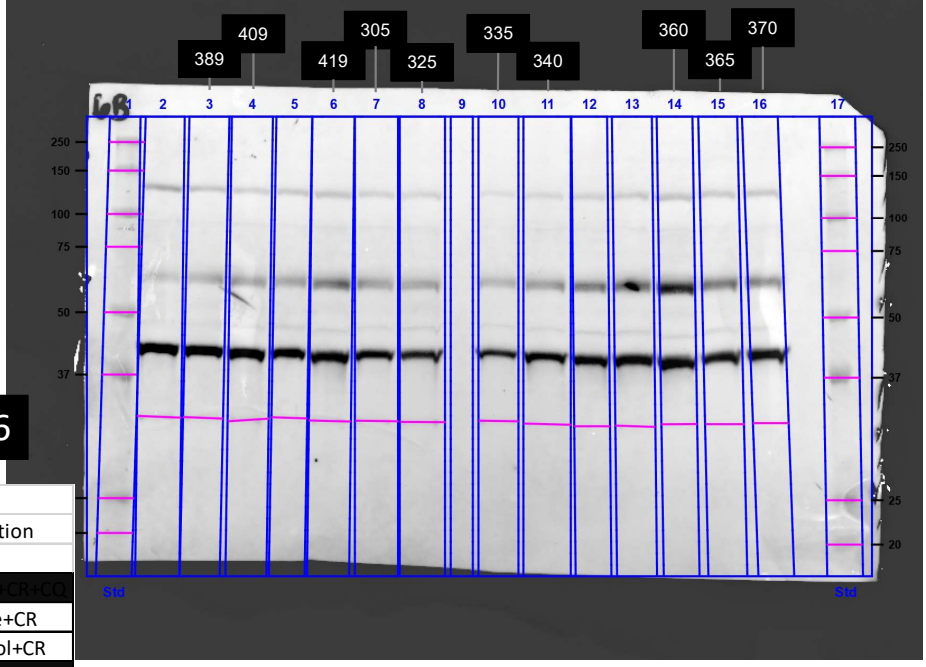

pRPS6

Blot 6			
Well	Sub#	Sex	Condition
1	Ladder		
2	384	Female	Chow+CR+CQ
3	389	Female	Obese+CR
4	409	Female	Control+CR
5	414	Female	DIO+CR+CQ
6	419	Female	Control
7	305	Male	Control
8	325	Female	Control
9	330	Female	Obese
10	335	Male	Obese+CR
11	340	Male	Control+CR
12	345	Male	DIO+CR+CQ
13	355	Female	DIO+CR+CQ
14	360	Female	Control+CR
15	365	Male	Control+CR
16	370	Male	Obese+CR
17	375	Male	Control
18	Ladder		

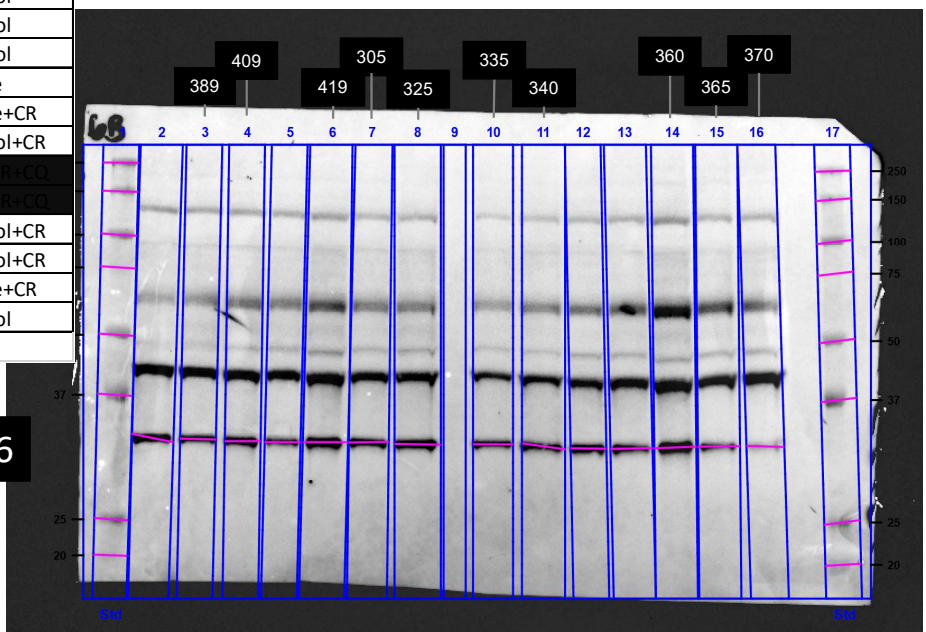

RPS6

*Blacked out cells are samples from another project that were run alongside these samples, but are not represented in the results of this manuscript.

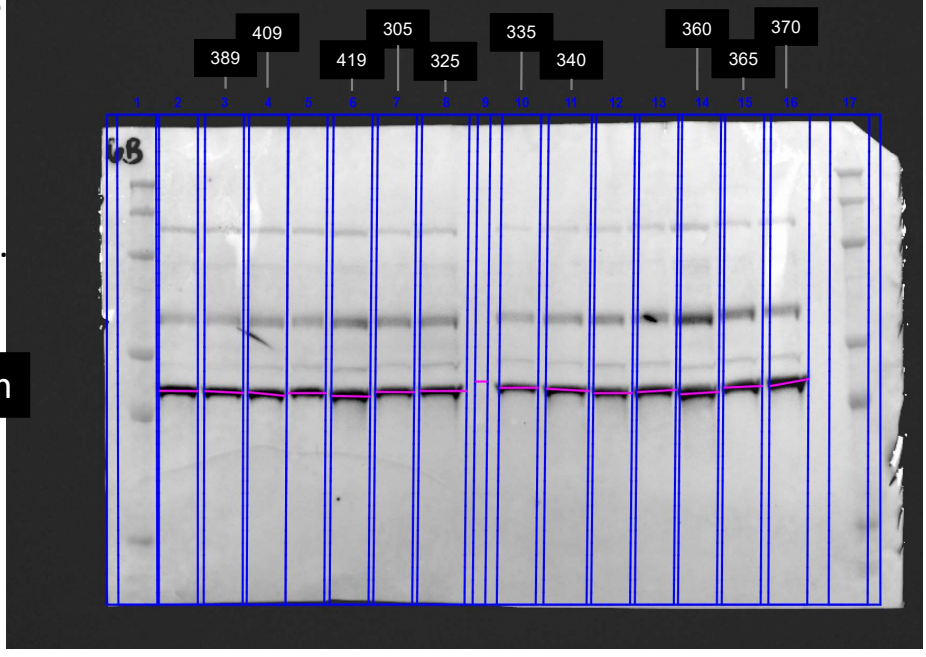

Actin

Blot 7A

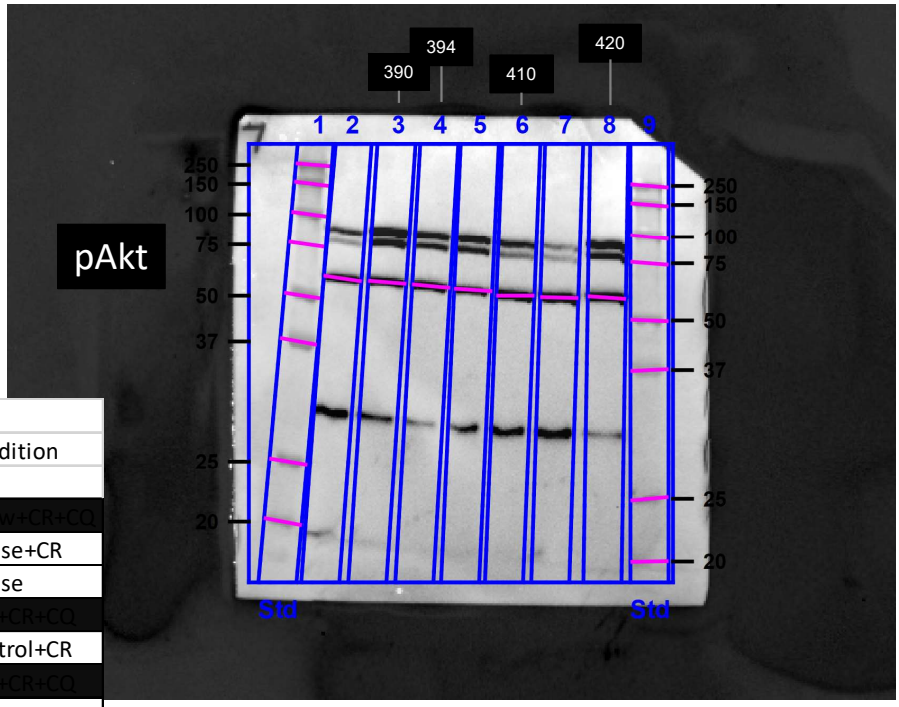

Blot 7			
Well	Sub#	Sex	Condition
1	Ladder		
2	385	Female	Chow+CR+CQ
3	390	Female	Obese+CR
4	394	Male	Obese
5	405	Male	DIO+CR+CQ
6	410	Female	Control+CR
7	415	Female	DIO+CR+CQ
8	420	Female	Control
9	Ladder		

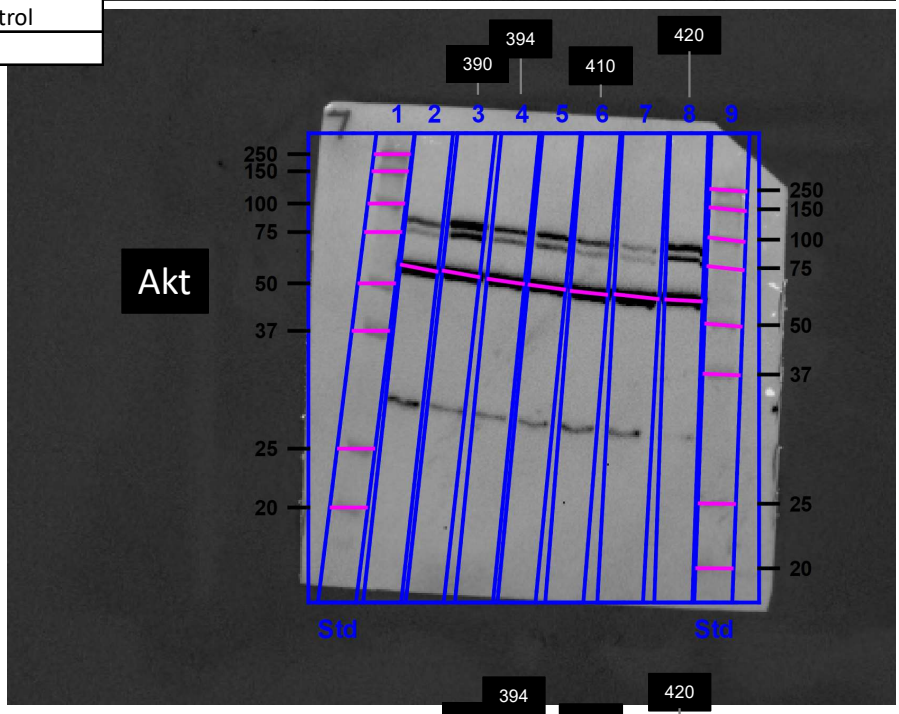

*Blacked out cells are samples from another project that were run alongside these samples, but are not represented in the results of this manuscript.

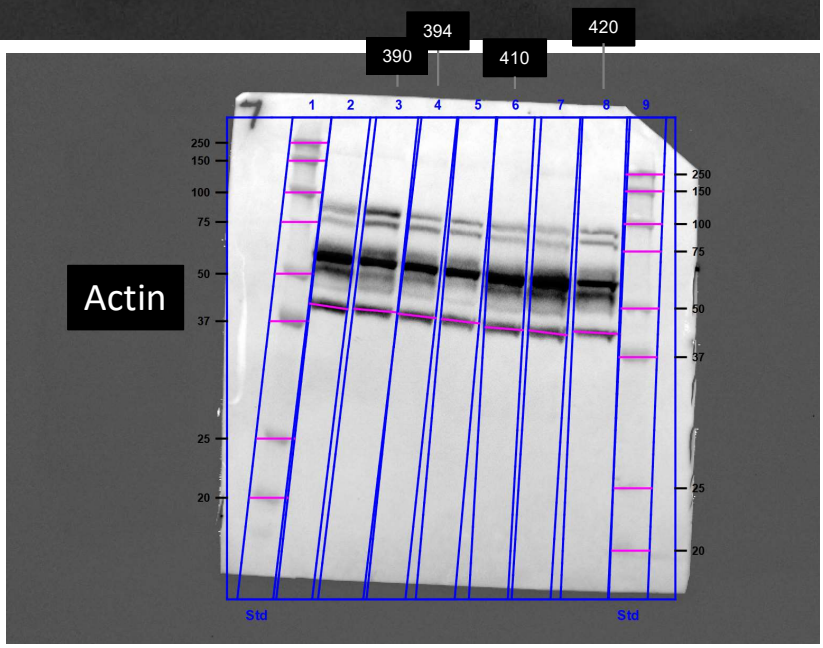

Blot 7B

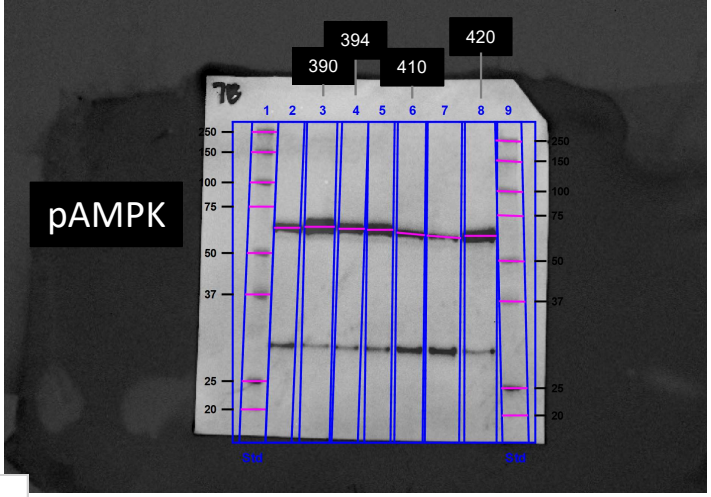

Blot 7			
Well	Sub#	Sex	Condition
1	Ladder		
2	385	Female	Chow+CR+CQ
3	390	Female	Obese+CR
4	394	Male	Obese
5	405	Male	DIO+CR+CQ
6	410	Female	Control+CR
7	415	Female	DIO+CR+CQ
8	420	Female	Control
9	Ladder		

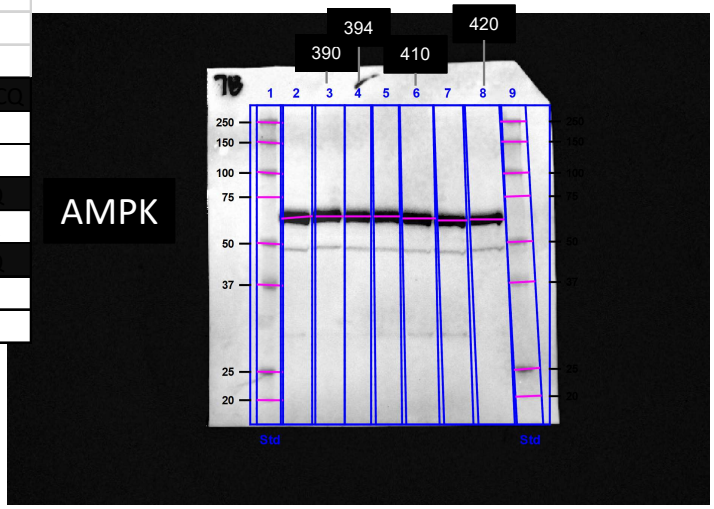

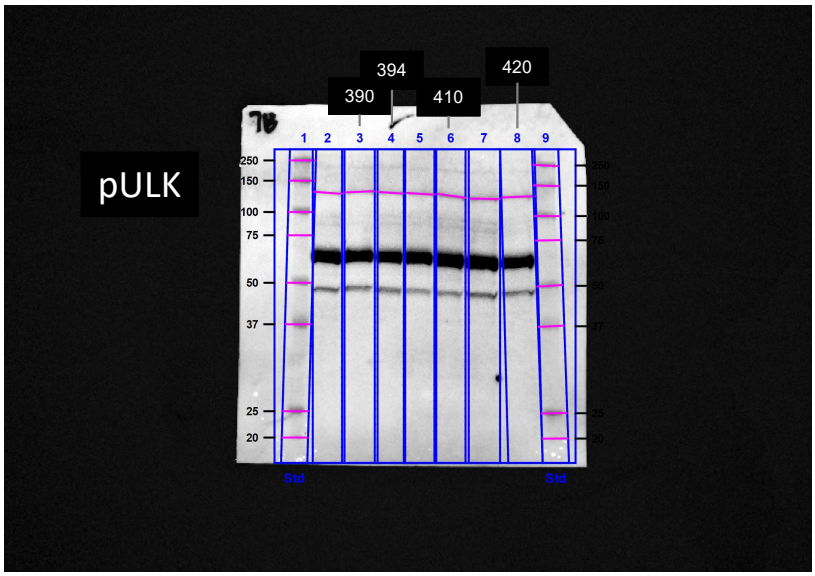

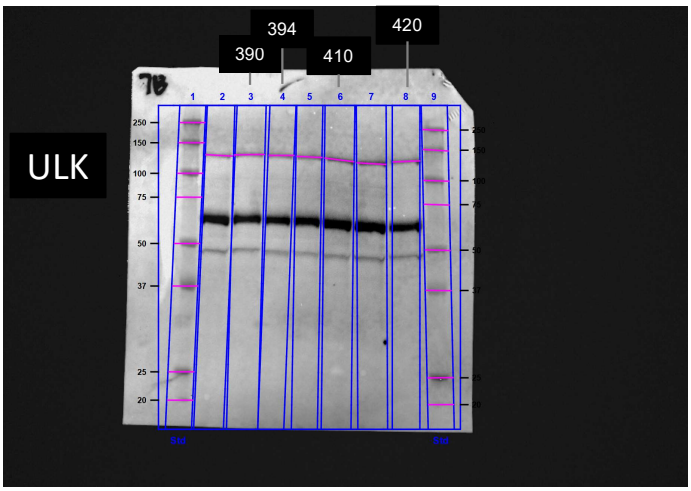

*Blacked out cells are samples from another project that were run alongside these samples, but are not represented in the results of this manuscript.

Blot 7C

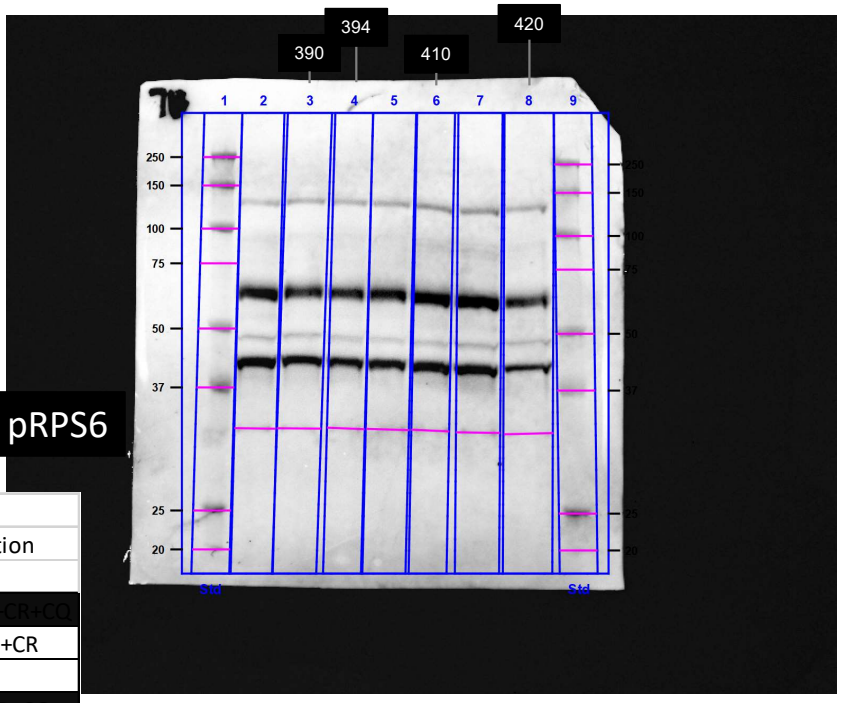

pRPS6

Blot 7			
Well	Sub#	Sex	Condition
1	Ladder		
2	385	Female	Chow+CR+CQ
3	390	Female	Obese+CR
4	394	Male	Obese
5	405	Male	DIO+CR+CQ
6	410	Female	Control+CR
7	415	Female	DIO+CR+CQ
8	420	Female	Control
9	Ladder		

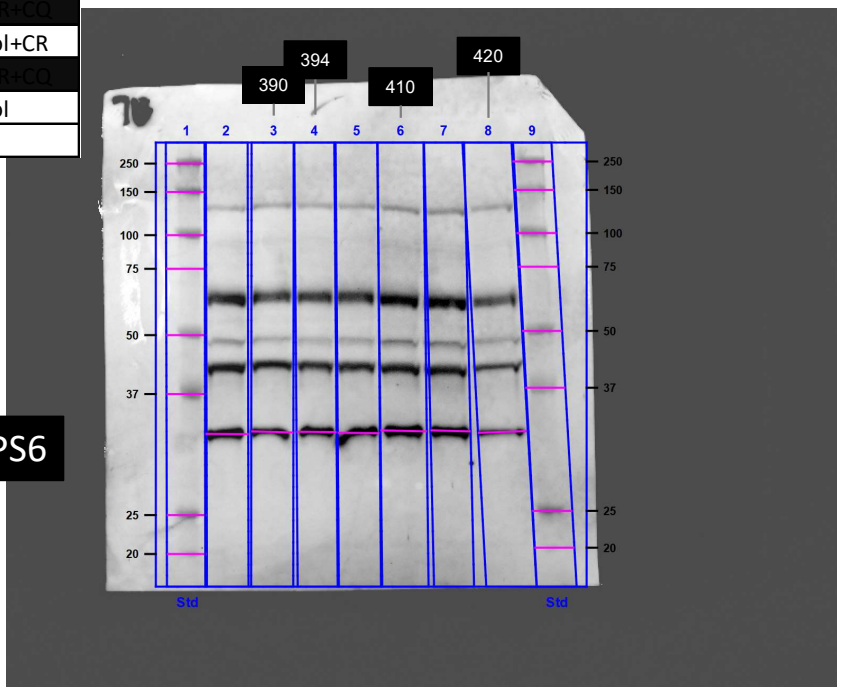

RPS6

*Blacked out cells are samples from another project that were run alongside these samples, but are not represented in the results of this manuscript.

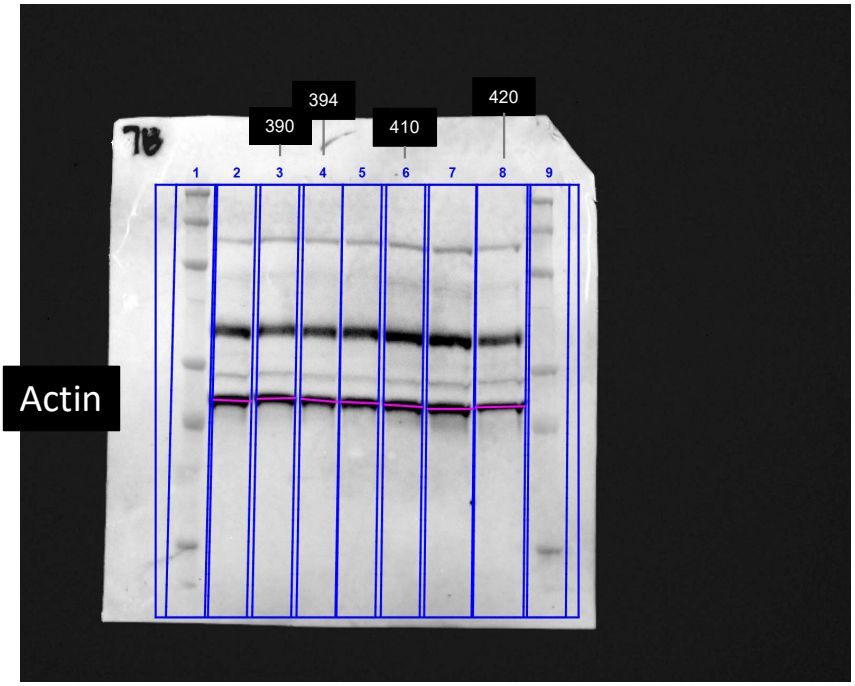

Actin
